# Alzheimer’s disease patient brain extracts induce multiple pathologies in novel vascularized neuroimmune organoids for disease modeling and drug discovery

**DOI:** 10.1038/s41380-025-03041-w

**Published:** 2025-05-02

**Authors:** Yanru Ji, Xiaoling Chen, Zhen Wang, Connor Joseph Meek, Jenna Lillie McLean, Yang Yang, Chongli Yuan, Jean-Christophe Rochet, Fei Liu, Ranjie Xu

**Affiliations:** 1 Department of Basic Medical Sciences, College of Veterinary Medicine, Purdue University, West Lafayette, IN 47907 USA; 2 Purdue Institute for Integrative Neuroscience (PIIN), Purdue University, West Lafayette, IN 47907 USA; 3 Borch Department of Medicinal Chemistry and Molecular Pharmacology, College of Pharmacy, Purdue University, West Lafayette, IN 47907 USA; 4 Departments of Structural Biology and Developmental Neurobiology, St. Jude Children’s Research Hospital, Memphis, TN 38105 USA; 5 Davidson School of Chemical Engineering, Purdue University, West Lafayette, IN 47907 USA; 6 Department of Neurochemistry, Inge Grundke-Iqbal Research Floor, New York State Institute for Basic Research in Developmental Disabilities, 1050 Forest Hill Road, Staten Island, NY 10314 USA

**Keywords:** Neuroscience, Stem cells

## Abstract

Alzheimer’s Disease (AD) is the most common cause of dementia, afflicting 55 million individuals worldwide, with limited treatment available. Current AD models mainly focus on familial AD (fAD), which is due to genetic mutations. However, models for studying sporadic AD (sAD), which represents over 95% of AD cases without specific genetic mutations, are severely limited. Moreover, the fundamental species differences between humans and animals might significantly contribute to clinical failures for AD therapeutics that have shown success in animal models, highlighting the urgency to develop more translational human models for studying AD, particularly sAD. In this study, we developed a complex human pluripotent stem cell (hPSC)-based vascularized neuroimmune organoid model, which contains multiple cell types affected in human AD brains, including human neurons, microglia, astrocytes, and blood vessels. Importantly, we demonstrated that brain extracts from individuals with sAD can effectively induce multiple AD pathologies in organoids four weeks post-exposure, including amyloid beta (Aβ) plaque-like aggregates, tau tangle-like aggregates, neuroinflammation, elevated microglial synaptic pruning, synapse/neuronal loss, and impaired neural network activity. Proteomics analysis also revealed disrupted AD-related pathways in our vascularized AD neuroimmune organoids. Furthermore, after treatment with Lecanemab, an FDA-approved antibody drug targeting Aβ, AD brain extracts exposed organoids showed a significant reduction of amyloid burden, along with an elevated vascular inflammation response. Thus, the vascularized neuroimmune organoid model provides a unique opportunity to study AD, particularly sAD, under a pathophysiological relevant three-dimensional (3D) human cell environment. It also holds great promise to facilitate AD drug development, particularly for immunotherapies.

## Introduction

Alzheimer’s Disease (AD) is a devastating neurodegenerative disorder and the most common cause of dementia, affecting more than 55 million individuals globally [1, 2]. Despite extensive efforts, most therapeutics have failed in clinical trials till now, with only limited effective treatments currently available [3–5]. AD is manifested with progressive cognitive decline and is characterized by multiple pathological hallmarks, including extracellular amyloid beta (Aβ) plaques, intracellular microtubule-associated tau neurofibrillary tangles (NFTs), neuroinflammation, synapse/neuronal loss, and brain atrophy [2, 4, 6]. To understand the mechanisms and develop therapeutics for AD, various animal models have been developed, significantly advancing our understanding of the disease [7–10]. However, most existing models focus on dominantly inherited familial AD (fAD), which is caused by genetic mutations in genes identified in individuals with fAD, including amyloid precursor protein (APP), presenilin1 (PSEN1), and presenilin2 (PSEN2) [7, 10, 11]. In contrast, models for sporadic AD (sAD), which do not involve highly penetrating genetic mutations and account for 95% of all AD cases, are severely limited [3, 10]. While both fAD and sAD share similar pathological hallmarks, such as Aβ plaques and NFTs, the etiology of sAD is still largely unknown and may involve more complicated genetic and environmental interactions [9, 10]. Thus, developing appropriate models for sAD without genetic mutations is critically needed. Moreover, significant species differences exist between humans and rodents. For instance, many repeat sequences in the genome and patterns of gene splicing are unique to humans [12]. These species differences may have profound impacts when modeling neurological disorders, particularly for AD, and may significantly contribute to the failures of many AD therapeutics that succeeded in pre-clinical animal studies but failed in clinical trials [3, 8, 12]. Therefore, it is critical to use human samples and develop human AD models that could recapitulate sAD features to advance our understanding of the disease mechanism and facilitate the development of therapeutic interventions.

Given the challenge of accessing and manipulating functional human brain tissues, human pluripotent stem cells (hPSCs), which include human embryonic stem cells (hESCs) and induced pluripotent stem cells (iPSCs), have emerged as valuable tools to utilize human cells for studying human neurological disorders, including AD [3, 13, 14]. The hPSC-based in vitro models, including two-dimensional (2D) and 3D models with pure human neural cells, are easy to manipulate and enable a high throughput for screening, making them valuable for modeling AD [3, 15–17]. The hPSC 2D model is useful for dissecting the impact of genetic and other factors on the individual cell types involved in AD at a basic functional level [18–26]. On the other hand, recent studies have also highlighted the value of hPSC 3D models, such as brain organoids and multiple cell type co-culture-based models, which incorporate various neural cell types and intricate cell-cell and cell-matrix interactions and preliminary neural structures, offering a more physiologically relevant environment to model diverse aspects of AD [27–35]. For example, fAD iPSC-derived brain organoids have successfully replicated AD pathological events, including Aβ aggregates and hyperphosphorylated tau, and facilitated the understanding of AD, such as the roles of APOE4 and C3 complement in AD [27, 29, 32, 33].

However, critical limitations persist, impeding these 3D models from effectively modeling AD. (1) Most research used simple organoids composed mainly of neurons with/without astrocytes [29–31, 36]. Although some studies have integrated microglia into organoids [37, 38], or incorporated blood vessels into 3D neural culture [39, 40], it is still a challenge to integrate multiple cell types, particularly microglia and blood vessels simultaneously within one organoid to reflect the complex pathophysiological environment of AD brains. (2) The current organoid models for AD study mainly focus on fAD, and these models usually take three to six months to exhibit AD pathologies [29, 33, 34, 41]. An intriguing recent study induced sAD-like phenotypes by exposing healthy organoids to serum to mimic the condition of blood-brain barrier (BBB) leakage, a risk factor for sAD [35]. However, a brain organoid model that contains multiple cell types affected in AD brains, capable of effectively recapitulating key AD pathological hallmarks of sAD within a relatively short time frame induced by an efficient trigger for modeling sAD, is still lacking.

To circumvent these limitations, in this study, we developed a vascularized neuroimmune organoid model for studying AD, particularly for sAD. This organoid model contains multiple cell types that are affected in human AD brains, including human neurons, astrocytes, microglia, and blood vessels. Since AD postmortem brain tissues contain proteopathic seeds, including Aβ and tau, that have the prion-like seeding activity to induce the counterpart normal protein to aggregate in animal models [42–46], we hypothesize that sAD postmortem tissue-derived brain extracts that contain both Aβ and tau seeds, can induce multiple AD pathologies in human cells in organoids, mirroring observations in sAD. Remarkably, our results demonstrated that organoids exposed to AD brain extracts successfully recapitulated multiple AD-like pathologies, including Aβ plaque-like aggregates, tau tangle-like aggregates, neuroinflammation, elevated microglial synaptic pruning, synapse/neuronal loss, but not organoids treated with vehicle control. Furthermore, we validated our organoids for drug discovery using Lecanemab, an FDA-approved anti-Aβ antibody for AD treatment and observed a significant alleviation of Aβ burden and potential side effects after treatment in the organoids that have been exposed to AD brain extracts. Thus, this vascularized neuroimmune organoid model presents a unique opportunity to study sAD and holds great promise to facilitate AD drug development.

## Results

### Generation and characterization of vascularized neuroimmune organoids

**Fig. 1:**
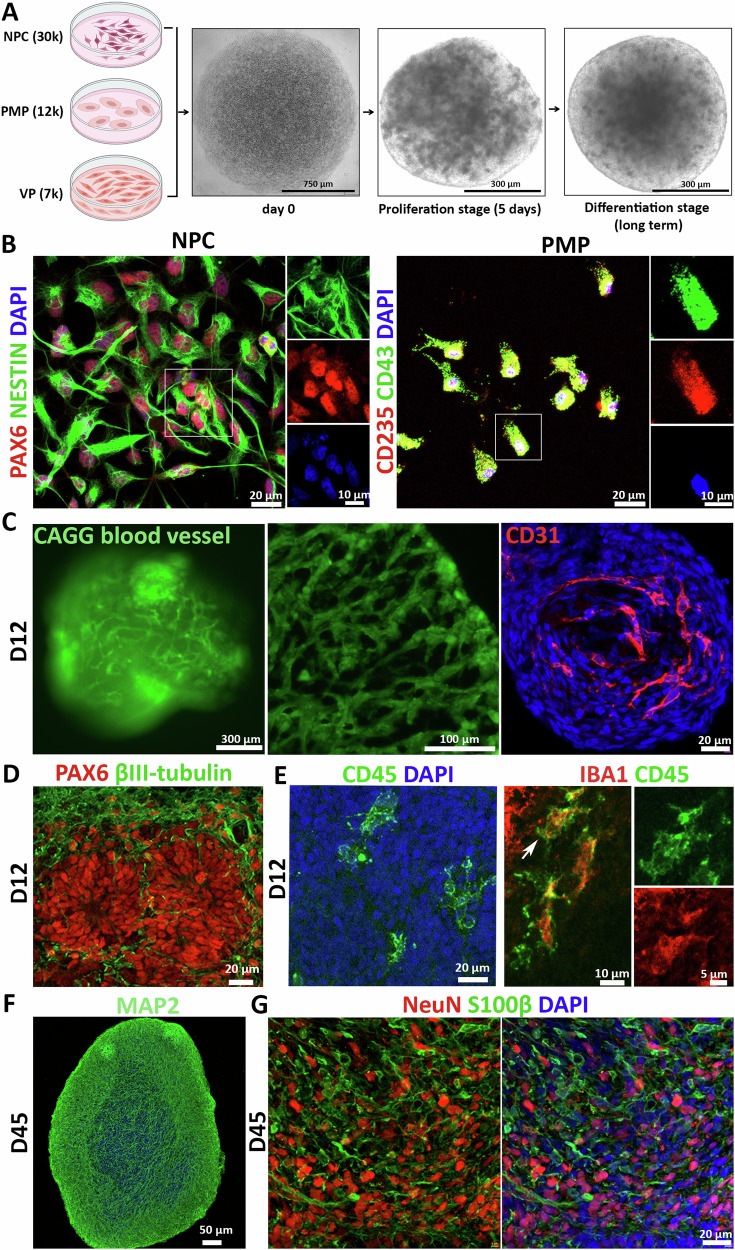
Generation and characterization of vascularized neuroimmune organoids. **A** Schematic image of the generation of vascularized neuroimmune organoids. hPSC-derived NPCs, PMPs, and VPs were co-cultured to form 3D spheroids. The proliferation stage lasted for five days before organoids were cultured in a differentiation medium. Scale bars, 750 or 300 µm as indicated. **B** Left panel, representatives of PAX6^+^ and NESTIN^+^ NPCs. Right panel, CD235^+^ and CD43^+^ PMPs. Scale bars, 20 or 10 μm as indicated. **C** Representatives of blood vessels in 12-day-old organoids. Left panel, CAGG-derived blood vessels in living organoids; Middle panel, sections of organoids with incorporated CAGG-derived blood vessels; Right panel, representative image of CD31 staining. Scale bars, 300, 100, or 20 μm as indicated. **D** Representative image of neural rosette stained with PAX6 and βIII-tubulin in 12-day-old organoids. Scale bar, 20 μm. **E** Characterization of microglia in 12-day-old organoids. Left panel, representative image of CD45^+^ microglia; Right panel, representative image of CD45^+^Iba1^+^ microglia. Scale bars, 20, 10, or 5 μm as indicated. **F** Representative image of MAP2 expression in 45-day-old organoids. Scale bar, 50 μm. **G** Representative images of NeuN^+^ neurons and S100β^+^ astrocytes in 45-day-old organoids. Scale bar, 20 μm.

To develop a brain organoid model that contains major cell types affected in human AD brains, building upon our previously established microglia-containing brain organoid model [47], we aim to introduce blood vessels into these organoids and develop a vascularized neuroimmune organoid model by co-culture of hPSC-derived neural progenitor cells (NPCs), primitive macrophage progenitors (PMPs), and vascular progenitors (VPs) under 3D conditions. To achieve this goal, we first generated NPCs and PMPs following our previous studies [37, 47, 48] (Fig. 1A). The identity of hPSC-derived NPCs was confirmed by co-expression of neural progenitor cell markers, PAX6 and NESTIN. PMPs were confirmed by co-expression of CD235, a marker for YS primitive hematopoietic progenitors [47], and CD43, a marker for hematopoietic progenitor-like cells [47] (Fig. 1B). VPs were generated using a published protocol with modifications detailed in Materials and Methods [49]. To generate brain organoids, the initial number was calibrated and co-cultured at 30,000 for NPCs, 12,000 for PMPs, and 7000 for VPs, based on our previous experience. These cells spontaneously assembled and formed organoids on day 1. Subsequent culture of the organoids was supplemented with mitogen fibroblast growth factor (bFGF) for 5 days to promote cellular proliferation (referred to as Proliferation stage). Thereafter, organoids were transitioned to a neural differentiation medium that contained neurotrophic factors, interleukin-34 (IL-34), vascular endothelial growth factor (VEGF), and other necessary supplements for long-term culture to support neuronal, microglial, and vascular maturation (referred to as Differentiation stage) (Fig. 1A). To ensure the genetic diversity and robustness of our findings, five different hPSC lines (four iPSC lines and one hESC line) from healthy individuals were used to derive progenitor cells. Please see Table S1.

To visualize the formation of blood vessels in living organoids, we incorporated VPs from an additional GFP^+^ hESC line (CAGG line). This allows us to track the vascular lineage cells by GFP signal. Under live cell imaging, clear and distinct branching structures were revealed in organoids on day 12 of culture (Fig. 1C, left panel). Organoid sections without immunostaining also displayed lumen-like structures (Fig. 1C, middle panel, and Fig. S1A and S1B). Subsequently, we stained organoids with CD31, an endothelial cell marker, and observed CD31^+^ cells in organoids (Fig. 1C, right panel). To further validate additional components of the vasculature, we performed staining in organoids containing GFP-tagged VPs with Collagen IV, a primary component of the basement membrane that supports endothelial cells and pericytes [50], and PDGFRβ, a marker for pericytes that are critical for providing structural support to endothelial cells [51]. Results suggested Collagen IV formed vascular basement membrane wrapping around GFP^+^ cells (Fig. S1C). PDGFRβ co-localized with GFP signals, indicating VPs also differentiated into pericytes (Fig. S1D). Together, these results confirmed the establishment of vascular structures within organoids.

On day 12, ventricular zone-like regions were seen in organoids, which contained PAX6^+^ progenitors and βIII-Tubulin^+^ immature neurons (Fig. 1D), mimicking the proliferative region in the developing cortical brain. To confirm PMPs differentiate into microglia at the differentiation stage, we first examined the expression of CD45 at day 12, which is a marker for all nucleated hematopoietic cells [47]. CD45^+^ cells exhibited a relatively even spatial distribution in organoids (Fig. 1E, left panel). Microglial identity was further confirmed by double staining of CD45 and IBA1, a canonical macrophage/microglia marker [47]. CD45 and IBA1 double staining showed ramified morphology, indicating healthy and functional microglia residing in organoids (Fig. 1E, right panel). Previous studies have shown that NPCs can mature into neuronal lineage cells, including neurons and astrocytes, in brain organoids [47, 52]. To confirm neural maturation in the organoids, we first stained MAP2, a marker for neuronal dendrites, and observed robust expression of MAP2 at Day 45 (Fig. 1F), suggesting NPCs efficiently differentiated into neurons. Further staining with NeuN, a mature neuron marker, and S100β, an astrocyte marker, revealed robust NeuN^+^ cells and S100β^+^ cells, demonstrating that NPCs differentiated into mature neurons and astrocytes during the differentiation stage (Fig. 1G). Taken together, we successfully developed vascularized neuroimmune organoids incorporating neurons, astrocytes, microglia, and vascular networks, which allow us to model the complex human brain environment.

### Sporadic AD (sAD) patient-derived brain extracts induce amyloid pathology in organoids

**Fig. 2:**
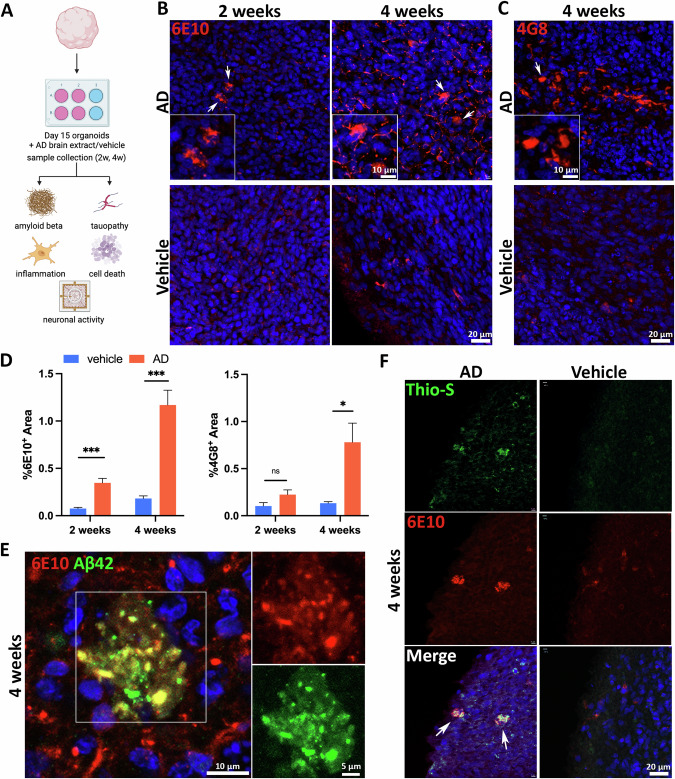
Sporadic AD patient-derived brain extracts induce amyloid pathology in organoids. **A** Schematic image of experimental design. **B** Representative images of 6E10 immunoreactivity in organoids at 2 or 4 weeks post-exposure to AD brain extracts or vehicle. Scale bars, 20 or 10 µm as indicated. **C** Representative image of 4G8 immunoreactivity in organoids at 2 or 4 weeks post-exposure to AD brain extracts or vehicle. Scale bars, 20 or 10 µm as indicated. **D** Graphs showing quantification of percentage area of 6E10^+^ signals and 4G8^+^ signals over time. *n* = 7 (6E10) or *n* = 4 (4G8) independent experiments from 4 or 3 hPSC lines, respectively. Each experiment used one hPSC line and contained 4–6 organoids. Data are presented as mean ± SEM. Unpaired t test with Welch’s correction, *p < 0.05, ***p < 0.001, ‘ns’ represents no significance. **E** Representative images of co-staining with 6E10 and Aβ42 in organoids at 4 weeks post-exposure to AD brain extracts. Scale bars, 10 or 5 µm as indicated. **F** Representative images of Thioflavin-S^+^ and 6E10^+^ structures in organoids at 4 weeks post-exposure to AD brain extracts or vehicle. Scale bar, 20 μm. The arrows indicate the co-localization of extracellular 6E10^+^ and Thioflavin-S^+^ plaque-like aggregates.

Our next aim is to enable the vascularized neuroimmune organoids to efficiently recapitulate key AD pathological features and facilitate the investigation of their interactions in studying sAD without genetic mutations. Prior studies have shown that human misfolded proteopathic seeds, such as Aβ or tau, can induce corresponding pathologies in transgenic mice engineered to express these human proteins [42–46]. Notably, recent studies suggested that Aβ seeds may also be able to transmit among humans. For instance, individuals who received Aβ-contaminated cadaver-derived pituitary growth hormone (c-hGH) during childhood later developed Aβ pathologies and cerebral amyloid angiopathy (CAA) [53–55]. Based on these findings, we hypothesized that AD individual-derived brain extracts that contain proteopathic seeds, including Aβ or tau, will induce AD pathologies in organoids as seen in human AD brains. To test our hypothesis and model sAD, we treated organoids with brain extracts from frozen temporal cortex tissues of sAD patients histopathologically confirmed to be at Braak Stages V-VI. sAD individual postmortem tissue-derived brain extracts (henceforth, the AD group) to model sAD. The corresponding vehicle (buffer) was used as control (henceforth, the vehicle group). In addition, age-matched healthy individual-derived brain extracts were also included. Organoids after 10 days of neuronal differentiation were exposed to AD brain extracts or the vehicle for two days, and samples were collected at 2 weeks or 4 weeks post-exposure. Subsequently, we examined AD pathological hallmark expression, including Aβ and tau pathologies, inflammation, and synapse/neuronal loss in organoids (Fig. 2A). To compare the effect of sex on the severity of pathologies, we included 3 male hPSC cell lines and 1 female hPSC line in the set of experiments.

To examine Aβ pathology characterized by extracellular Aβ aggregates in organoids, we utilized 6E10 and 4G8 antibodies, which can detect the amino acid residues 3–8 or 17–24 of Aβ, respectively [56]. Two weeks post brain extract or vehicle exposure, a modest number of weakly diffused 6E10 and 4G8 signals were detected in both the AD and vehicle groups (Figs. 2B and S2). Interestingly, only the AD group exhibited intraneuronal Aβ aggregates, as indicated by the presence of discrete, punctate 6E10 signals around nuclei (Fig. 2B, top panel). To exclude the possibility that aggregates were caused by other components contained in the brain extracts other than toxic protein seeds, age-matched healthy individual-derived brain extracts were included as the healthy control group. Similar to the vehicle group, diffuse but not aggregated signals were observed in healthy control brain extract-treated organoids (FFig. S3). Since 6E10 can detect both Aβ and intraneuronal amyloid precursor protein (APP) [56], the diffuse 6E10 and 4G8 signals might represent the endogenous APP. To confirm the presence of intraneuronal Aβ aggregates in the AD group, we stained organoids with MOAB-2 antibody, which can specifically label intraneuronal Aβ, but not APP [56]. MOAB-2^+^ cells were only detected in the AD group, confirming the formation of intraneuronal Aβ aggregates (Fig. S4).

Remarkably, at 4 weeks post AD brain extract exposure, multiple dense extracellular plaque-like aggregates were detected in the AD group but not the vehicle group (Fig. 2B) or healthy control brain extract-treated organoids (Fig. S3). These extracellular aggregates were consistently identified in the AD group by 4G8 staining (Fig. 2C). Quantitative analysis showed that compared to the vehicle group, the AD group demonstrates a significant escalation of Aβ burden, as indicated by the positive areas of 6E10 and 4G8 (Fig. 2D). A comparison of 6E10^+^ areas between male and female samples exhibited no significant difference at 4 weeks post-AD brain extract exposure (Fig. S5A). To confirm the composition of extracellular Aβ aggregates, we performed double staining of organoids with 6E10 with another antibody Αβ42, which primarily targets Aβ42 [57]. The overlapped punctate signals of 6E10 and Αβ42 were observed from extracellular regions in the AD group, suggesting that extracellular Aβ aggregates contain Aβ42 (Fig. 2E). The partial overlapping signals between 6E10 and Αβ42 suggest that 6E10 may also detect other forms of Aβ and extracellular aggregates consisting of both Αβ42 and additional Aβ species. Moreover, to assess Aβ plaque formation, we conducted double staining of organoids with 6E10 with a dye, Thioflavin-S, which binds to β-sheet structure in protein aggregates, including plaques and tangles [58]. Consistently, the vehicle group showed some diffuse intraneuronal 6E10 signals, which were Thioflavin-S negative; only the AD group exhibited the co-localization of strong extracellular 6E10 and Thioflavin-S signals, reinforcing the presence of plaque-like aggregates in the AD group (Fig. 2F, top panel). Collectively, these findings demonstrated that AD brain extracts containing proteopathic Aβ seeds can induce Αβ pathology, including Aβ plaque-like aggregates, in healthy brain organoids within a 4-week post-exposure time frame.

### Sporadic AD (sAD) patient-derived brain extracts induce tau pathology in organoids

**Fig. 3:**
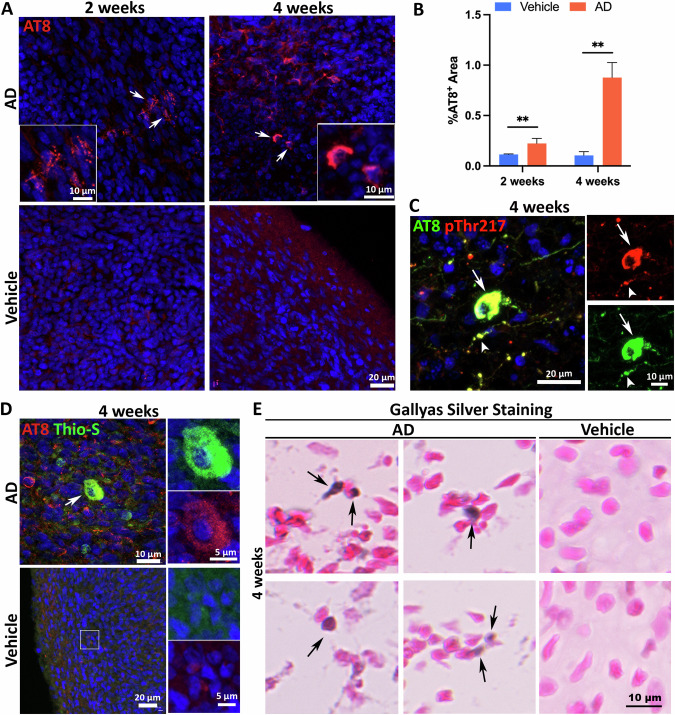
Sporadic AD patient-derived brain extracts induce tau pathology in organoids. **A** Representative images of AT8^+^ cells in organoids at 2 or 4 weeks post-exposure to AD brain extracts or vehicle. Scale bars, 20 or 10 μm as indicated. **B** Graphs showing quantification of AT8^+^ cells over time. *n* = 7 independent experiments from 4 hPSC lines, each experiment used one hPSC line and contained 4–6 organoids. Data are presented as mean ± SEM. Unpaired t test with Welch’s correction, **p < 0.01. **C** Representative images of AT8 and pThr217 double staining in organoids at 4 weeks post AD brain extract exposure. The arrow and arrowhead indicate the co-localized AT8 and pThr217 signal in the soma and processes, respectively. Scale bars, 20 or 10 μm as indicated. **D** Representative images of AT8 and Thioflavin-S labeled hyperphosphorylated tau aggregates in organoids at 4 weeks post-exposure to AD brain extracts. The arrow indicates the co-localization of intracellular AT8^+^ and Thioflavin-S^+^ tangle-like aggregates. Scale bars, 20, 10, or 5 μm as indicated. **E** Representative images of Gallyas silver staining of organoid at 4 weeks post-exposure to AD brain extracts or vehicle. The arrows indicate intracellular tangle-like structures. Scale bar, 10 μm.

Tau pathology, caused by the abnormal accumulation of the microtubule-associated protein tau, is a major pathological hallmark of AD. In human AD brains, tau undergoes abnormal hyperphosphorylation and other modifications, which convert it into a pathological protein with prion-like seeding activity and form neurofibrillary tangles (NFTs) [59, 60]. Given that the AD brain extracts applied in this study contained tau seeds (Fig. S6), we wonder whether the AD brain extract can induce tau pathology in our organoid model. To examine tau pathology, we utilized a widely used antibody, AT8, which can detect phosphorylated tau [45]. By 2 weeks post-exposure, the AD group presented with puncta-like, AT8^+^ intraneuronal signals (Fig. 3A, top panel), potentially indicating the formation of aggregates in neurons. Notably, 4 weeks post-AD brain extract exposure, the intensity and quantity of condensed AT8 signals increased significantly, suggesting further formation of tau aggregates (Fig. 3A, top panel). In contrast, the vehicle group displayed no such phosphorylated tau aggregates at either time point (Fig. 3A, bottom panel), nor did healthy control brain extract-treated organoids (Fig. S7). Quantification revealed a substantial increase in AT8^+^ areas in the AD group compared to the vehicle group (Fig. 3B), suggesting that AD brain extract was sufficient to induce tau pathology in organoids. Additionally, different from the condensed AT8 signals after AD brain extract exposure, weak and diffuse axonal AT8^+^ signals were observed in organoids at 2 weeks post-exposure to the vehicle and healthy control brain extracts (Fig. 3A, S7, and S8), aligning with prior studies that identified transient phosphorylated tau in neurons during development [53, 61]. Confirmation of tau aggregate was further achieved through double staining of AT8 and another hyperphosphorylated tau marker, pThr217 —a widely used clinical diagnostic marker. Results showed significant overlap and pathology-related morphology, suggesting that the current model recapitulates the tau phosphorylation at multiple sites (Fig. 3C). The utilization of Thioflavin-S and AT8 co-staining marked Thioflavin-S^+^ and AT8^+^ double-positive cells exclusively in the AD group (Fig. 3D). To further confirm the formation of tau tangle-like structures, we performed Gallyas Silver Staining, a commonly used method for detecting tau tangles [62], and observed the formation of NFT-like structures in the AD group at four weeks post-exposure (Fig. 3E). In addition, while there is an increased trend of AT8^+^areas in the female samples after 4 weeks post-AD brain extract exposure, no statistical difference was found (Fig. S5B). In all, our findings demonstrated that sAD brain extract can successfully induce tau pathology, including tau tangle-like aggregates, in healthy brain organoids.

### The AD neuroimmune organoids recapitulate neuroinflammation, phagocytosis of Αβ, and excessive microglial synaptic pruning

**Fig. 4:**
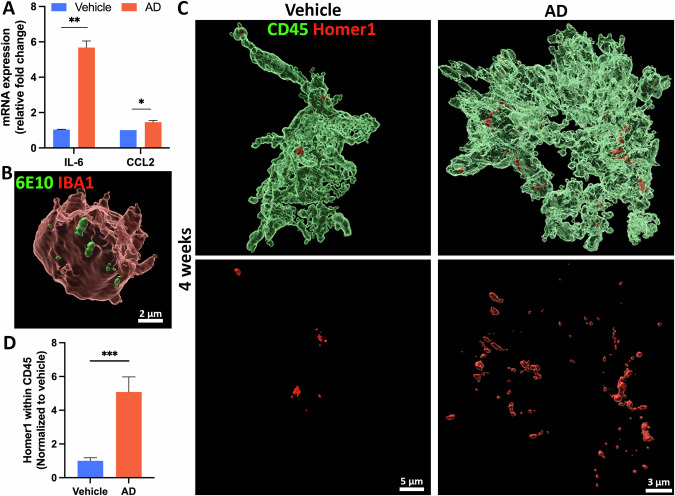
The AD neuroimmune organoids recapitulate neuroinflammation, phagocytosis of Αβ, and excessive microglial synaptic pruning. **A** Graph showing mRNA expression levels of *IL-6* and *CCL2* in organoids from 4–6 weeks post-exposure to AD brain extract or vehicle, determined via qRT-PCR. *n* = 3 independent experiments from two hPSCs lines, each experiment used one hPSC line and contained 4–6 organoids. Unpaired student’s t test, *p < 0.05, **p < 0.01. **B** 3D reconstructed image showing microglia phagocytizing Aβ. Organoid sections from the AD group treated with AD brain extracts were stained with IBA1 and 6E10. Scale bar, 2 μm. **C** 3D reconstructed image showing microglial synaptic pruning. Sections from organoids at 4 weeks post-exposure to the vehicle or AD brain extracts were stained with Homer1 and hCD45. Scale bars, 5 or 3 μm as indicated. **D** Graph showing quantification of Homer1 puncta engulfment within CD45^+^ microglia for the vehicle and AD groups. The phagocytic activity of microglia was quantified by dividing the Homer1 puncta volume by the microglia volume. *n* = 4 independent experiments from three hPSC lines, each experiment used one hPSC line and contained 4–6 organoids. Data are presented as mean ± SEM. Unpaired t test with Welch’s correction, ***p < 0.001.

In the AD environment, accumulating amyloid aggregates trigger the activation of microglia and astrocytes, and stimulate neuroinflammation by releasing inflammatory factors [63–65]. Since neuroimmune organoids contain microglia and astrocytes, to determine whether neuroimmune organoids can replicate neuroinflammation in AD brains, we measured the mRNA expression of the pro-inflammatory cytokine, IL-6, and chemokine, CCL2, by qRT-PCR in the organoids at four to six weeks post-exposure to AD brain extracts or vehicle treatments. As shown in Fig. 4A, compared to the vehicle group, the AD group exhibits a significant increase in mRNA levels of IL-6 and CCL2, demonstrating that AD neuroimmune organoids recapitulated neuroinflammation. In addition, microglia play a beneficial role in AD by phagocytosis and removal Aβ. To examine whether microglia in the AD group display this function, we conducted double staining of organoids with 6E10 and IBA1. 3D-reconstructive images from the Imaris software confirmed microglial engulfment of Aβ in the AD group, aligning with previous evidence of microglial involvement in Aβ clearance in AD [66, 67] (Fig. 4B).

One significant detrimental role of microglia in AD is the abnormal removal of synaptic materials [68]. We subsequently evaluated whether human microglia in AD neuroimmune organoids could recapitulate this phenomenon. Organoids were stained with CD45 and Homer1 (a post-synaptic marker) at four weeks post-exposure to AD brain extracts or vehicles. The 3D-reconstructed images revealed that microglia pruned synaptic materials (Fig. 4C). Quantification of the volume of Homer1 puncta within the volume of CD45 indicated a significant increase of microglial pruning in the AD group (Fig. 4D). In summary, our results indicated that neuroimmune organoids can recapitulate neuroinflammation, as well as dynamic microglial functions in an AD-like 3D environment, which mirrors in vivo human microglia behaviors in the presence of AD pathologies.

### The AD neuroimmune organoids recapitulate synapse/ neuronal loss and impaired neural activity

**Fig. 5:**
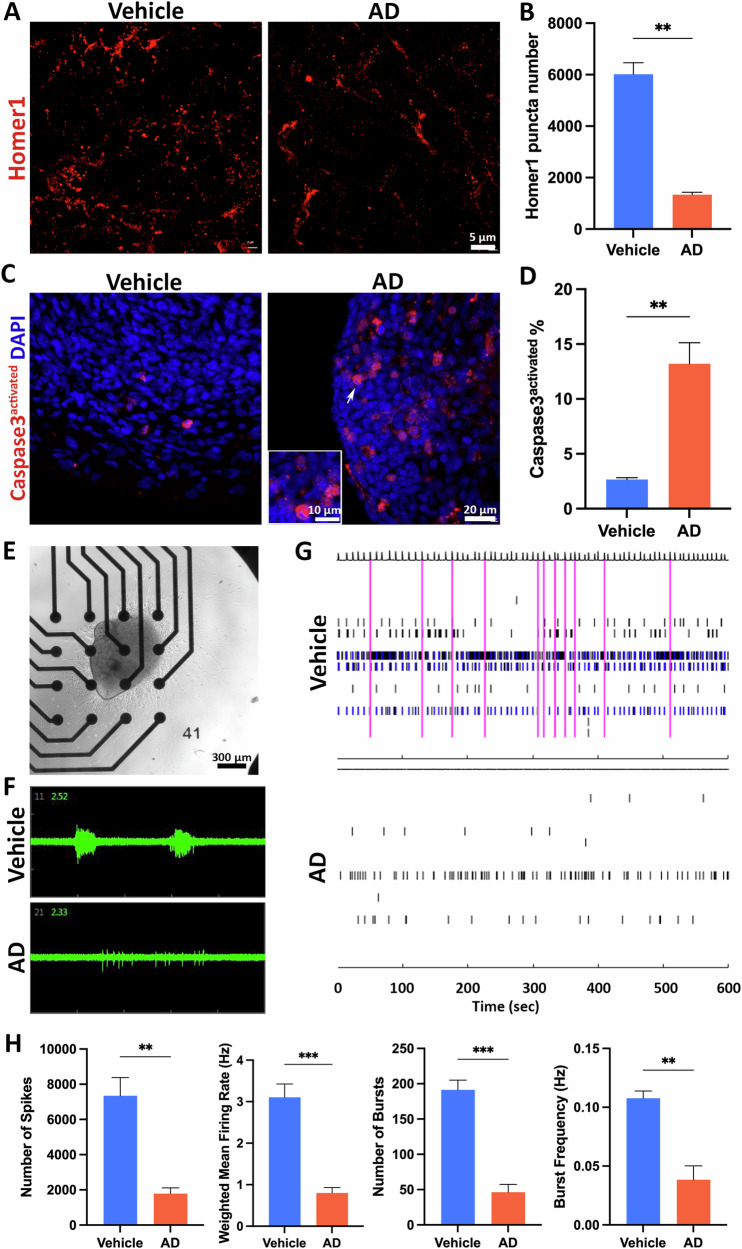
The AD neuroimmune organoids recapitulate synapse/ neuronal loss and impaired neural activity. **A** Representative images of Homer1 staining in the vehicle and AD groups at 4 weeks post-exposure to the vehicle or AD brain extracts, respectively. Scale bar, 5 μm. **B** Graph showing quantification of Homer1 puncta number per 2500 μm^2^. *n* = 4 independent experiments from three hPSC lines, each experiment used one hPSC line and contained 4–6 organoids. Data are presented as mean ± SEM. Unpaired t test with Welch’s correction, **p < 0.01. **C** Representative images of activated Caspase-3 staining in vehicle and AD groups at 4 weeks post-exposure to the vehicle or AD brain extracts, respectively. Scale bars, 20 or 10 μm as indicated. **D** Graph showing quantification of the percentage of Caspase-3^+^ cells. *n* = 4, four independent experiments from three hPSC lines, each experiment used one hPSC line and contained 4–6 organoids. Data are presented as mean ± SEM. Unpaired t test with Welch’s correction, **p < 0.01. **E** Representative image of organoid attached to an MEA plate. Scale bar, 300 μm. **F** Representative plots of spikes/bursts recorded within 2 s from a single electrode in proximity to organoids from the vehicle or AD group. **G** Representative spike raster plots generated from MEA recording raw data from the vehicle group and AD group. **H** Graphs showing quantification of MEA parameters. *n* = 4 independent experiments from two hPSC lines, each experiment used one hPSC line and contained 4–6 organoids. Data are presented as mean ± SEM. Unpaired t test with Welch’s correction, **p < 0.01, ***p < 0.001.

Synapse/neuronal loss is the key feature of AD [6, 69] and AD animal models are often limited to recapitulating neuronal loss [70–72]. To investigate whether brain organoids challenged by AD brain extracts could recapitulate this process, we first examined synaptic integrity by staining organoids with Homer1. Quantitative analysis of the number of Homer1 puncta per 2500 μm^2^ revealed a reduction in the AD group (Fig. 5A, B), indicating synapse loss in the AD group. Subsequently, we stained organoids with active Caspase3 to assess neuronal death in organoids. Compared to the vehicle group, a significant increase in active Caspase3^+^ cells was detected in the AD group, demonstrating elevated neuronal death in the AD group (Fig. 5C, D).

To further understand whether synapse/neuronal loss causes functional defects, we assessed the neural network activity in the organoids employing the MEA assay (Fig. 5E–G). Spontaneous neural activities were recorded and analyzed in organoids that were seeded on electrodes and cultured for two additional weeks after four weeks post-AD brain extract or vehicle treatment. Our data revealed a reduced number of spikes, mean firing rate, and number of bursts in the AD group (Fig. 5H), suggesting impaired neural network activity in the AD group. Together, these results indicated an environment conducive to synapse/neuronal loss, accompanied by compromised neural network activity in AD neuroimmune organoids.

### Proteomics analysis highlights disrupted pathways in AD neuroimmune organoids

**Fig. 6:**
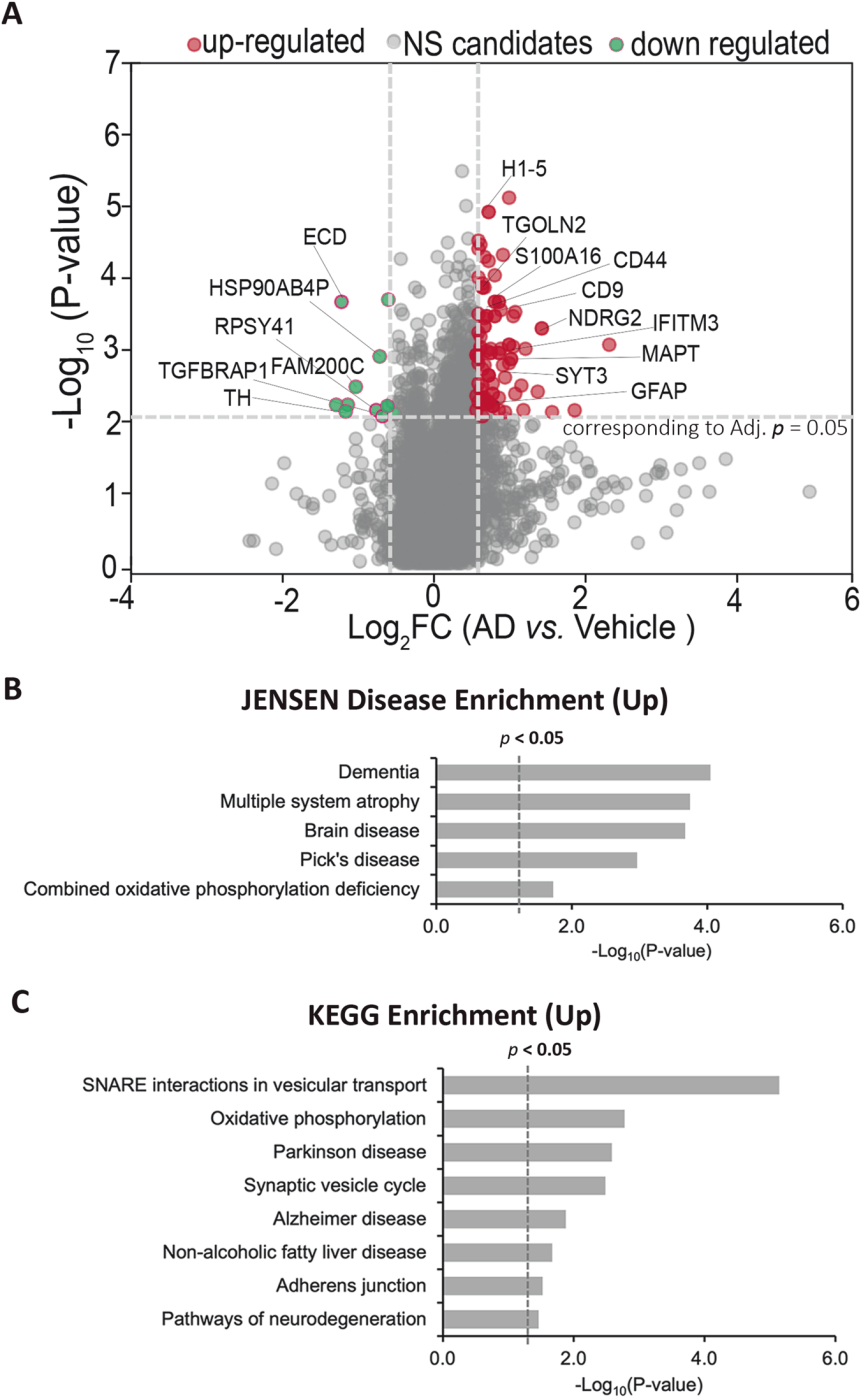
Proteomics analysis highlights disrupted pathways in AD neuroimmune organoids. **A** Volcano plot displaying DEPs between AD group and vehicle group organoids (FC ≥ 1.5, *p* < 0.05, and FDR < 1%). A total of 87 proteins were identified, with 76 proteins upregulated and 11 proteins downregulated. **B** Top 5 pathways identified via JENSEN Disease Enrichment analysis of the 76 identified upregulated proteins. **C** Top 8 pathways identified via KEGG Enrichment analysis of the 76 identified upregulated proteins.

To obtain molecular features and more comprehensively characterize AD neuroimmune organoids, we performed quantitative proteomics analysis to compare organoids from the control and AD groups at four weeks post-treatment of either vehicle or AD brain extracts. Each group included three samples, with three to four organoids per sample. Around 5800 proteins across both sample groups were identified. Applying criteria of FC ≥ 1.5 and adjusted *p* < 0.05, we identified 87 differentially expressed proteins (DEPs) in the AD organoids, comprising 76 upregulated and 11 downregulated proteins compared to the vehicle group (Fig. 6A and Tables S4 and S5). Notably, proteins such as CD44, GFAP, and MAPT- previously recognized as significantly upregulated proteins in AD human brain tissues using proteomics analysis and are considered as potential biomarkers for AD [73, 74]- were among the upregulated DEPs, suggesting AD organoids align with the established AD characteristics.

To assess the functional pathways of the DEPs, we performed pathway enrichment analysis using the upregulated DEPs. Jensen DISEASES analysis identified dementia as the top 1 annotated pathway (Fig. 6B), confirming the relevance of our findings. KEGG enrichment analysis further showed that the upregulated DEPs were enriched in pathways such as SNARE interactions in vesicular transport, synaptic vesicle cycle, Alzheimer’s Disease, adherens junction, pathways of neurodegeneration, consistent with previous findings on the molecular signature of AD patients (Fig. 6C) [75]. Additionally, MGI Mammalian Phenotype Enrichment analysis highlighted pathways, such as abnormal miniature excitatory currents, abnormal hippocampus CA3 region morphology, abnormal psychological neovascularization, increased macrophage cell number, and abnormal dendrite morphology (Fig. S9), which is also consistent with AD features and our characterization of cellular dysfunction in AD neuroimmune organoids.

Consistent with previous findings, GFAP levels were elevated, indicating astrocyte activation and neuroinflammation in the AD group organoids (Fig. 6A). The expression of CD44 and CD9 was upregulated (Fig. 6A), suggesting a shift in the microglial profile toward an AD-associated phenotype [76–78]. We also identified the upregulation of IFITM3 (Fig. 6A), a protein elevated in the brain tissue of a subset of late-onset AD patients, which is associated with increased neuroinflammation and correlated with enhanced γ-secretase activity and amyloid beta production [79].

Among the downregulated proteins, excluding those unreported in AD, many proteins have been reported to be decreased in AD in previous studies, such as HSP90AB4P, TH, TMEM70, and TGFBRAP1 (Fig. 6A). As an example, HSP90AB4P, as a member of the heat shock proteins (HSPs), which are molecular chaperones playing a crucial role in regulating protein aggregation, HSP90 genes were found to have reduced expression in AD patients [39, 80]. A smaller number of downregulated DEPs were identified compared to the upregulated DEPs. This may be due to a greater accumulation of proteins in AD organoids, consistent with observations from proteomics studies using human AD brain samples [81–84]. Additionally, more samples may be needed to enhance the depth of proteomics analysis in the future.

To further evaluate how the molecular signature of AD neuroimmune organoids resembles that of patients, we compared the 76 upregulated DEPs with publicly available proteomics datasets from human AD brain tissues on the NeuroPro website (https://neuropro.biomedical.hosting/) [75]. 28 out of 76 upregulated proteins overlapped with previously identified upregulated proteins in brain samples of pre-clinical/mild cognitive impairment/clinical stage AD patients (analyzed by https://bioinformatics.psb.ugent.be/webtools/Venn/) (Table S6). The overlapping proteins are enriched in Jensen Disease Enrichment pathways, including Dementia, Multiple system atrophy and Brian disease (Fig. S10), highlighting the resemblance of AD neuroimmune organoids to the characteristics observed in patients. Overall, our proteomics data indicate that the AD neuroimmune organoids successfully replicate the major disrupted pathways and molecular signatures of AD observed in patients, corroborating our findings on cellular dysfunction in these organoids.

### Anti-Aβ antibody lecanemab relieved amyloid burden in AD neuroimmune organoids

**Fig. 7:**
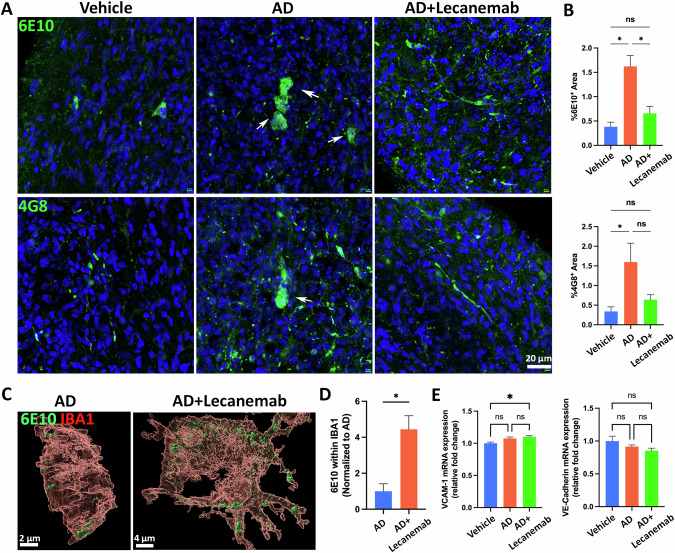
Anti-Aβ antibody Lecanemab relieves amyloid burden in AD neuroimmune organoids. **A** Representative images of 6E10 and 4G8 staining from the vehicle group, AD group, and AD+Lecanemab group. The arrows indicate 6E10^+^ or 4G8^+^ aggregates. The Scale bar, 20 μm. **B** Graphs showing quantification of 6E10^+^ and 4G8^+^ area. *n* = 3, three independent experiments from three hPSC lines, each experiment used one hPSC line and contained 4–6 organoids. Data are presented as mean ± SEM. One-way ANOVA, *p < 0.05, ‘ns’ represents no significance. **C** Representative images of 3D reconstructed figures showing the internalization of Aβ by microglia. Organoids from the AD group and AD+Lecanemab group were stained with IBA1 and 6E10. Scale bars, 4 or 2 μm as indicated. **D** Graph showing quantification of microglial phagocytosis of Aβ. *n* = 3 independent experiments from three hPSC lines, each experiment used one hPSC line and contained 4–6 organoids. Data are presented as mean ± SEM. Unpaired two-tailed t test with Welch’s correction, *p < 0.05. **E** Graphs showing mRNA expression level of *VCAM-1* and *VE-Cadherin* in organoids generated from ApoE4/4 C112R hiPSC cell line, determined via qRT-PCR in the vehicle, AD, and AD+Lecanemab groups. *n* = 4 independent experiments, each experiment contained 3–4 organoids. One-way ANOVA, *p < 0.05, ‘ns’ represents no significance.

Since AD organoids can recapitulate AD pathologies, we wonder whether the organoid model could be used for AD drug discovery. Lecanemab, an FDA-approved humanized monoclonal Aβ antibody for treating early AD, targets and neutralizes toxic Aβ, facilitating their clearance from the brain [85–87]. Given that our organoids displayed Aβ pathology at four weeks post-exposure to AD brain extracts, we treated these neuroimmune organoids with Lecanemab for 2 consecutive weeks to evaluate its efficacy in our organoids. Staining with 6E10 and 4G8 post-treatment indicated that Lecanemab alleviated the amyloid burden in AD organoids (Fig. 7A, B). It has been suggested that Lecanemab may induce phagocytosis and removal of Aβ by microglia in human brains [88, 89]. Therefore, we investigated the involvement of microglia in Lecanemab-mediated Aβ clearance in our organoids. Through staining with 6E10 and IBA1 and subsequent observation via 3D reconstructive images, we noted that microglia appeared to phagocytize Aβ in the organoids. Interestingly, Lecanemab-treated organoids exhibited increased phagocytosis of Aβ (Fig. 7C, D).

Lecanemab is associated with side effects in some patients, such as amyloid-related imaging abnormalities (ARIA), which are characterized by brain swelling and vascular impairment-related bleeding detectable via MRI [90]. To examine whether our organoid can recapitulate the potential side effects of Aβ antibody treatments, we assessed the impact of Lecanemab in the organoid on vascular integrity and the vascular immune response, which are disrupted in the case of Aβ antibody treatments and associated with ARIA [91]. Given the significantly higher risk of developing ARIA in patients carrying the ApoE ε4 allele, we generated vascularized neuroimmune organoids using a ApoE4/4 hiPSC line. We first measured the mRNA expression levels of VCAM-1, a vascular-immune-related protein that has been shown to be upregulated in aged brains and cerebral microbleeds that contribute to an inflammatory profile [92–94]. The qPCR results indicated a slight increase in VCAM-1 mRNA expression levels following treatment of the organoids with AD brain extracts. This expression level further increased in the Lecanemab-treated group, suggesting the activation of brain endothelial cells [92, 93], and an elevated vascular inflammation response (Fig. 7E, left panel). We also measured the expression of VE-Cadherin, a critical transmembrane protein that maintains the junctions between endothelial cells and promotes vascular integrity [95]. The mRNA expression level of VE-Cadherin exhibited a trend of downregulation in the AD group and the Lecanemab-treated group compared to the vehicle group organoids (Fig. 7E, **right panel**), suggesting a potential disruption of endothelial adherent junctions and vascular integrity. Overall, our results suggested that Lecanemab could relieve the Aβ burden in AD organoids, which might involve phagocytosis of Aβ by microglia. Lecanemab treatment can also partially recapitulate the side effects, such as impaired vascular integrity and altered vascular immune response. Consequently, our organoids demonstrated potential as a promising in vitro platform for testing AD therapeutics by evaluating both their efficacy and potential side effects.

## Discussion

Effectively modeling sAD to recapitulate multiple pathologies, and developing platforms for drug discovery, have been challenging due to the complexity of the disease and the significant species differences between human and animal models. In this study, we presented a novel vascularized neuroimmune organoid model composed of human neurons, astrocytes, microglia, and blood vessels. Upon exposure to sAD individual-derived extracts, the organoid successfully recapitulated multiple pathological features in sAD, including Aβ plaque-like aggregates, neurofibrillary tangle-like aggregates, neuroinflammation, microglial synaptic pruning, synapse/neuronal loss, and impaired neural network activity. Furthermore, by validating the FDA-approved anti-Aβ antibody Lecanemab, the organoid model demonstrates its potential as a platform for in vitro AD drug development, particularly for immunotherapies.

In contrast to other hiPSC-based AD brain organoid models, our vascularized neuroimmune organoid model has significant advantages that enable us to model AD more efficiently and test AD therapeutics. **(1)** The new organoid model incorporates multiple key cell types that are affected in human AD brains under a pathophysiologically relevant 3D human cell-centric environment. Our organoid simultaneously includes neurons, astrocytes, microglia, and vasculatures, which enables a more comprehensive understanding of cell-cell interactions and cell-type-associated pathological events in AD, such as neuroinflammation and microglial synaptic pruning, which are absent in most AD organoid models (Fig. 4). In addition to AD, the new organoid model also enables the study of the interactions of different cell types during neurodevelopment and other neurological disorders. **(2)** The new organoid model can effectively develop multiple AD pathologies within a relatively short time frame, enabling the study of the interactions of AD pathologies, which is suitable for studying AD, particularly sAD. Previous AD organoid models were usually derived from fAD iPSC lines, which need long-term culture to replicate the AD pathologies. For instance, studies have indicated that it took at least 5 months to exhibit amyloid plaque-like and neurofibrillary tangles-like aggregates [33], or 90 days to observe Aβ aggregates and hyperphosphorylated tau protein [28], in fAD iPSC-derived brain organoids. Additionally, a recent study using healthy iPSC line-derived organoids to model sAD through serum exposure required 3–4 months of culture to recapitulate Aβ aggregates and hyperphosphorylated tau [35]. However, in our organoids, at 4 weeks post-exposure to AD brain extracts, we successfully recapitulated multiple relatively mature AD pathologies, including Aβ plaque-like aggregates and tau tangle-like aggregates, neuroinflammation, and synapse/neuronal loss, in around 1.5-month-old organoids. With a similar strategy, it is possible to efficiently model fAD by exposing fAD-derived brain extracts to fAD iPSC-derived organoids. **(3)** The new organoid model is useful to serve as an in vitro platform to advance AD drug development, particularly antibody-based therapeutics (Fig. 6), because the organoids contain microglia and can efficiently recapitulate multiple AD pathologies, and microglia are expected to be a major cell component to degrade the antibody-binding protein aggregates, such as Aβ.

Our results demonstrate that AD brain extracts can efficiently induce multiple AD pathologies in neuroimmune organoids, enabling the study of their interaction during disease progression of sAD. AD pathologies are known to occur at different stages of the disease [4], Aβ accumulation is generally considered an early event that triggers inflammation and contributes to tau pathology, occurring approximately two decades before clinical symptoms. Excessive microglial synaptic pruning is also critical in the early stages, while synapse/neuronal loss becomes more pronounced in the later stages, correlating with the emergence of clinical symptoms [96]. A major goal of the current study is to recapitulate multiple AD pathologies during disease progression, which will allow the future study of the interactions of these pathologies for AD to advance our understanding of disease mechanisms, particularly for sAD. To model sAD, we first generated the neuroimmune organoids from hPSC lines from healthy individuals without the need for familial AD mutations. Previous studies demonstrated that proteopathic seeds, such as Aβ or tau, have prion-like activity to induce corresponding pathologies in transgenic mice engineered to express these human proteins [42–46], and Aβ seeds also can induce Aβ pathologies in patients [53–55]. However, it is unknown whether proteopathic seeds can induce pathologies in human brain organoids. We hypothesized that AD individual-derived brain extracts that contain proteopathic seeds, including Aβ or tau, will induce AD pathologies in organoids as seen in human AD brains. To achieve our goal and test our hypothesis, we exposed neuroimmune organoids to brain extracts derived from frozen temporal cortex tissues of sAD patients histopathologically confirmed to be at Braak Stages V-VI. Our results demonstrated that four weeks after exposure, the organoids exhibited multiple pathologies associated with AD, including Aβ and tau pathologies, inflammation, microglial synaptic pruning, and synapse/neuronal loss. These pathologies in organoids span early-stage processes like microglial synaptic pruning and late-stage features such as synapse/neuronal loss at 4 weeks post-exposure to AD brain extracts. Thus, these organoids likely mimic the progression of sAD, transitioning from early/middle to late stages, allowing for the evaluation of therapeutic interventions targeting early stages; however, future studies are needed to more precisely define the associated disease stages.

We propose that the neuroimmune organoids can recapitulate multiple pathologies for two main reasons. First, the proteopathic seeds in brain extracts from sAD patients (i.e., likely, a combination of Aβ and tau aggregates) may have high seeding activity, enabling the induction of Aβ and tau pathologies in our organoid model with high potency [97]. Second, the complex cellular composition of our organoids, which include neurons, astrocytes, microglia, and blood vessels, further facilitates the development of these features, particularly microglia and related neuroinflammation, absent in the previously mentioned organoids. For instance, the buildup of the protein aggregates initiates an inflammatory response and microglial phagocytic function, mimicking the disease progression as seen in patients. While not all pathological changes occur simultaneously in every individual with sAD, our organoid model provides a valuable platform to explore these interactions in a feasible manner. However, the variations in brain extracts derived from different patients, disease stages, and brain regions may result in discrepancies in the type, quantity, and activity of proteopathic seeds, which might influence the severity and types of pathologies induced in the organoids. It will be beneficial to characterize the brain extracts using proteomics and other methods before using them. To minimize variability, potential strategies in the future may include using extracts from multiple patients with consistent brain regions and disease stages or using controllable synthetic or recombinant seeds despite their potential for reduced seeding activity.

The AD neuroimmune organoids can recapitulate Aβ and tau pathologies. We first observed intraneuronal Aβ accumulation, as indicated by punctate-like high-intensity 6E10^+^ signals (Fig. 2B), at 2 weeks post AD brain extract exposure. By the fourth week, these accumulations had progressed to extracellular Aβ plaque-like structures, indicated by the colocalization of 6E10 and Thioflavin-S (Fig. 2B, C, and F), suggesting a developing pattern from intraneuronal aggregation to extracellular deposition. This pattern is in line with the findings in AD and Down Syndrome individuals, where intraneuronal amyloid aggregates were found in brain regions vulnerable to dementia, such as the hippocampus and entorhinal cortex, preceding the formation of extracellular plaques [98–102]. Similarly, organoids demonstrated puncta-like aggregates of hyperphosphorylated tau 2 weeks post AD brain extract exposure and exhibited tangle-like structures by the fourth week post-exposure, indicated by the Thioflavin-S and Gallyas Silver Staining (Fig. 3A, B, and D).

On the other hand, our results provide direct evidence that proteopathic seeds, not only Aβ but tau, can spread among human cells and tissue-like 3D structures, and induce AD-like Aβ and tau pathologies. This aligns with the clinical observations that Aβ contaminated c-hGH can cause Aβ pathologies and CAA in humans over decades [54, 55, 63]. This also corroborates the concept of the prion-like property of AD proteopathic proteins and the possible transmission of AD between humans by accidents in medical or surgical procedures, raising awareness to prevent iatrogenic human transmission of AD.

Neuroinflammation and synapse/neuronal loss are known to appear along the progression of AD [103]. In the early stages of the disease, stimuli like extracellular Aβ activate neuroinflammatory responses that may recruit microglia for Aβ clearance [63, 64]. Consistent with this, in our organoids, mRNA expression levels of inflammatory cytokines increased in the AD group (Fig. 4A). Furthermore, Aβ signals were detected inside of microglia, indicating microglial phagocytosis and clearance of Aβ in organoids (Fig. 4B). Additionally, consistent with previous studies showing excessive microglia-mediated synaptic pruning in AD animal models [68, 96], we observed a significant microglia-dependent synaptic removal (Fig. 4C, D) and a significant decrease in synaptic materials (Fig. 5A, B) in AD organoids. Additionally, elevated neuronal death was confirmed by the activated Caspase-3 signaling increment in the AD group (Fig. 5C and D). Although studies have found neuronal hyperactivity in the presence of Aβ pathology [104], in vivo research has proven that tau dominates Aβ when both are present in transgenic mouse models [105, 106]. In agreement with these observations, our MEA assay of organoids revealed impaired neural network activity (Fig. 5E–H), with fewer spikes and bursts recorded compared to the vehicle group, corroborating previous findings from animal studies [105].

The neuroimmune organoids are particularly valuable for testing AD therapeutics, especially immunotherapies, as they contain multiple cell types (including microglia) and exhibit AD-like pathologies. The recent FDA-approved anti-Aβ monoclonal antibodies, Lecanemab and Donanemab, underscore the tremendous potential of antibody-based immunotherapies for tackling AD [5, 107–109]. However, species differences may hinder the translational efficacy of preclinical animal models in clinical trials, highlighting the urgent need for human models to test these immunotherapies before clinical trials. **(1)** Recent cryo-EM findings have uncovered species-specific aggregate structures of proteopathic proteins in AD [110]. Aggregate structures observed in AD animal models are often not present in individuals with AD [111]. This discrepancy potentially impedes our assessment of the efficacy of the therapeutics and contributes to translation failures. **(2)** Antibody-based therapeutics targeting toxic proteins, such as anti-Aβ antibodies, are usually tested in animals. Before moving to clinical trials, successful antibody candidates undergo a humanization process to avoid immune rejection, such as by replacing the murine IgG with the human version [112]. However, the humanization process may substantially compromise the efficacy of the antibodies and even cause side effects [113]. Thus, direct testing of these human antibodies in human models is crucial. **(3)** Antibody-targeted protein aggregates, such as Lecanemab-binding Aβ protofibrils, are hypothesized to be recognized and removed by microglia, the immune cells in the brain [87–89]. However, there are no suitable human models available that can recapitulate AD pathologies and contain human microglia for directly testing these human versions of antibody-based therapeutics. Our organoids contain multiple cell types, including microglia, and develop AD-like pathologies that offer a unique platform to address this limitation. To assess whether our organoid model could serve as an ideal in vitro platform for testing AD drugs, we validated Lecanemab in our organoids. At 4 weeks post-exposure to AD brain extracts, we treated our AD organoids with Lecanemab for two weeks and observed a significant decrease in amyloid burden. Additionally, we also observed an increased microglial engulfment of Aβ in the Lecanemab-treated group (Fig. 6A–D), providing direct evidence that human microglia are involved in the Lecanemab-mediated Aβ removal. Although the current organoid model cannot fully recapitulate ARIA, qPCR results indicate an increase in VCAM-1 mRNA level and a trend towards a modestly decreased VE-Cadherin mRNA level after Lecanemab treatment (Fig. 6E), suggesting potential ARIA-associated vascular immune responses and impaired vascular integrity after treatment. These molecular-level insights may facilitate the prediction of potential side effects when the model is utilized for drug testing. Thus, the neuroimmune organoids not only model the efficacy of immunotherapies (Fig. 6A–D) but also partially represent their potential side effects (Fig. 6E), highlighting their promise as an effective in vitro platform for testing AD therapeutics.

Sex differences play a critical role in AD, with approximately two-thirds of AD individuals being female, and women exhibiting a faster AD pathology and cognitive decline [114–116]. In our study, while the female iPSC-derived organoids showed a trend towards greater tau pathology, no statistical differences in Aβ and tau pathologies were observed when compared to organoids derived from male hiPSC lines (Fig. S5). This may be attributed to several factors: (1) an insufficient culture period for organoids to exhibit sex differences. Previous studies have indicated that sex differences have a more pronounced effect on functional impairment, particularly during the later stages of pathology development [114]. A total culture duration of 4 weeks post-brain extract exposure might not be sufficient to reveal significant differences between female and male organoids. (2) In our study, we utilized one female and 3 male hiPSC lines to evaluate the impact of sex differences. The incorporation of additional female iPSC lines with diverse genetic backgrounds is warranted for future studies to evaluate sex differences. (3) Beyond sex chromosomes, sex hormones may also contribute to the different characteristics of disease progression in men and women [117]. However, current organoid models lack the complexity required to account for the effects of sex hormones. Thus, incorporating multiple female iPSC lines, extending culture time, and introducing sex hormones will be beneficial in future studies aimed at evaluating the impact of sex differences on AD pathologies.

While the inclusion of blood vessels improves the overall health of the organoids to better model the complexity of the brain environment, we did not detect the presence of CAA, a common vascular pathology in AD where Aβ fibrils are deposited along the cerebral vasculatures [69], using iPSC lines carrying the APOE3/3 or APOE4/4 variants (data not shown). In our current study, all iPSC lines were from healthy donors. It might be helpful to incorporate additional sAD cell lines in future research, which might more efficiently recapitulate AD-like pathologies. In particular, using sAD iPSC lines from AD donors with CAA, along with an extended culture period post-AD extract exposure, might be beneficial in modeling CAA. In addition, a critical challenge for iPSC-based brain organoid models is the relatively immature state of human cells [118]. Recent studies, including ours, have developed chimeric brain models by transplanting iPSC-derived organoids or neural cells into animal brains, where human cells can survive and mature as animals age under the conducive physiological in vivo environment [37, 48, 119, 120]. It will be promising to transplant our organoids into animal brains and test whether our organoids can better model AD features in the context of aged human cells within the host brain in the future. Overall, our innovative vascularized neuroimmune organoids present unique opportunities to model sAD and advance drug discovery for AD.

## Material and methods

### Culture and quality control of hPSC lines

Five different hiPSC lines, KOLF2.1, ND2, GCaMP, ApoE4/4 C112R and UTY1, as well as one hESC cell line CAGG, were used in this study (Supplementary Table S1). The hiPSCs were maintained under feeder-free conditions and cultured on hESC-qualified Matrigel (Corning) coated dish in mTeSR plus media (STEMCELL Technologies). The hiPSCs were passaged at approximately 70% confluency with ReLeSR media (STEMCELL Technologies).

### NPC generation and culture

Small molecule-based protocols were applied when generating NPCs, as in our previous studies [48, 121]. Neural differentiation in the embryoid bodies (EBs) was induced by dual inhibition of SMAD signaling. In summary, EBs were cultured in neural induction medium composed of DMEM/F12 (HyClone) and 1 × N2 Supplement (Thermo Fisher Scientific) supplied with inhibitor SB431542 (2 μM, Stemgent) and noggin (50 ng/mL, Peprotech) for 6 days before plating on growth factor-reduced Matrigel (BD Biosciences) coated plates. EBs were cultured with neural induction medium supplied with laminin (1 μg/mL, Corning) for 7 days, and then NPCs in the form of neural rosettes were manually isolated from surrounding cells. Isolated NPCs were expanded for 5–7 days, depending on the confluency, in NPC medium, which is composed of a 1:1 mixture of Neurobasal (Thermo Fisher Scientific) and DMEM/F12, supplemented with 1 × N2, 1 × B27-RA (Thermo Fisher Scientific), FGF2 (20 ng/mL, Peprotech), human leukemia inhibitory factor (hLIF, 10 ng/mL, Millipore), CHIR99021 (3 μM, Biogems), SB431542 (2 μM), and ROCK inhibitor Y-27632 (10 μM, Tocris).

### PMP generation and culture

PMPs were generated as previous studies [37, 122]. Briefly, embryoid bodies (EBs) were generated from hiPSCs and induced to yolk sac EBs (YS-EBs) by adding bone morphogenetic protein 4 (BMP4, 50 ng/mL, Peprotech; to induce mesoderm), vascular endothelial growth factor (VEGF, 50 ng/mL, Peprotech; endothelial precursors), and stem cell factor (SCF, 20 ng/mL, Miltenyi Biotech; Hematopoietic precursors) into mTeSR1 media (STEMCELL Technologies) for 5 days. Next, YS-EBs were plated into dishes and cultured with PMP medium composed of interleukin-3 (IL-3, 25 ng/mL, Peprotech) and macrophage colony-stimulating factor (M-CSF, 100 ng/mL, Peprotech) in XVIVO media (Lonza). Human PMPs emerged into the supernatant 2–3 weeks after plating and were continuously produced for more than 3 months.

### VP generation and culture

VPs were generated using a published protocol with minor modifications [49]. In brief, hiPSCs were dissociated to form aggregates in a low-attachment plate and differentiated into mesoderm with CHIR99021 (12 μM, Peprotech) and BMP4 (30 ng/mL) in a 1:1 mixture of Neurobasal and DMEM/F12, supplemented with 1 × N2, 1 × B27-RA for 3 days. Then, vascular lineage induction was performed with VEGF (100 ng/mL) and forskolin (2 μM) for 2 days. Cell aggregates were then embedded into growth factor-reduced Matrigel, cultured with VEGF (100 mg/mL), FGF2 (100 ng/mL), and 15% FBS (Gibco) in XVIVO media for 5 days to allow vessel sprouting. Cell aggregates were dissociated by TrypLE into single cells, which served as the VP for the following organoid generation.

### Brain extract preparation and tau seeding activity confirmation

To obtain 10% (w/v) brain extracts, frozen temporal cortex tissues of histopathologically confirmed AD cases with Braak Stages V-VI from the Brain Tissue Resource Center, McLean Hospital, Belmont, MA, USA, were prepared in homogenization buffer (20 mM Tris–HCl, pH 8.0, 0.32 M sucrose, 10 mMβ-mercaptoethanol (β-ME), 5 mM MgSO 4, 1 mM EDTA, 10 mM glycerophosphate, 1 mMNa 3VO4, 50 mM NaF, 2 mM benzamidine, 1 mM 4-(2-aminoethyl) benzenesul-fonyl fluoride hydrochloride (AEBSF), and 10 μg/ml each aprotinin, leupeptin, and pepstatin), and centrifuged at 10,000 × g for 30 min, as previously reported [123, 124]. Tau seeding activity of brain extracts was confirmed (Fig. S5), using in vitro seeded tau aggregation assay, in which HEK-293T cells expressing HA-tau_151–391_ were treated with brain extracts and lysed in RIPA buffer. Seeded HA-tau_151–391_ aggregates were yielded by ultracentrifugation and analyzed by immuno-blots, as previously reported [123, 124].

### Organoid assembly, culture, brain extract treatment, and drug treatment

To generate vascularized immune-brain organoids, 30,000 NPCs, 12,000 PMPs, and 7000 VPs were co-cultured for organoid self-assembly. Organoids were cultured in a 1:1 mixture of NPC medium (1:1 mixture of Neurobasal and DMEM/F12, supplemented with 1 × N2, 1 × B27-RA, FGF2 (20 ng/mL)) and PMP medium for 5 days to allow proliferation, and were transferred to ND medium, which is composed of a 1:1 mixture of Neurobasal and DMEM/F12, supplemented with 1 × N2, 1 × B27-RA, BDNF (10 ng/mL, Peprotech), GDNF (10 ng/mL, Peprotech), L-Ascrobic acid (200 nM, Sigma Aldrich), c-AMP (1 μM, Sigma Aldrich), IL-34 (100 ng/mL, Peprotech), M-CSF (25 ng/mL), TGF-β1 (50 ng/mL, Peprotech). After 10 days of differentiation, vehicle or brain extracts derived from AD patients, or age-matched healthy individuals (2 mg/ml), were added to the culture medium (10 μL/mL) for two days. The organoids were collected at 2 weeks or 4 weeks post-exposure to brain extracts or the vehicle. Four weeks post exposure to brain extracts, Lecanemab (Sellekchem, Cat#A3112) was added to medium at a concentration of 10 μg/mL. Samples were collected after 2 weeks of Lecanemab treatment.

### Immunohistochemistry and cell counting

Organoids were fixed with 4% paraformaldehyde and then cryo-sectioned at 15 μm thickness for immunostaining. The tissues were blocked with a blocking solution (5% goat or donkey serum in PBS with 0.2% Triton X-100) at room temperature for 1 hr. Primary antibodies were diluted in the same blocking solution and incubated at 4 °C overnight. Sections were washed with PBS and incubated with secondary antibodies for 1 h at room temperature. Sections were then washed with PBS and were mounted with anti-fade Fluoromount-G medium containing 1,40,6-diamidino-2-phenylindole dihydrochloride (DAPI) (Southern Biotechnology). Antibodies used were listed in Supplementary Table S2. For Thioflavin-S staining, sections were washed with 70 and 80% ethanol for 1 min, respectively, followed by 15 min of incubation of 0.05% freshly prepared and filtered Thioflavin-S solution. Samples were then washed with 80 and 70% ethanol sequentially for 1 min, and finally rinsed with water. Images were captured with a Zeiss LSM 900 confocal microscopy. Image analysis was performed using Fiji (NIH). Relative fluorescence intensity was presented as normalized value to the vehicle group. Cells, plaques, or synapses were counted with Fiji. At least three fields of each organoid were chosen randomly to count after Z projection. The data are repeated three to four times (*n* = 3 or 4) from 3 hPSC lines, each experiment used one hPSC line and contained 4–6 organoids.

### Gallyas silver staining

The Gallyas silver staining method was used to stain the Tau tangles [56], details are included in Supplementary materials. Slides were washed in distilled water for one minute and transferred immediately to alkali silver iodide for 15 min. Next, slides were washed for 1 min in 2% oxalic acid, followed by three washes in distilled water for 1 min. Then the slides were incubated in alkaline silver iodide solution for 2–5 min followed by three rinsing in 0.5% acetic acid for 1 min each. Then slides were incubated in physical developer solution for 15–20 min The developer was made fresh before use in a 1:1 ratio (solution A: solution B). Next, the samples were washed in 0.5% acetic acid and 1% sodium thiosulfate for 5 min each. Subsequently, samples were washed in distilled water for five minutes, and incubated in 0.5% gold chloride solution, and sodium thiosulfate for 5 min each. Finally, the samples were washed in distilled water for five minutes, counterstained with nuclear fast red, and mounted using Permount solution.

### RNA isolation and qPCR

Total RNA isolation was performed with TRIzol Reagent (Invitrogen) and complementary DNA was prepared with SuperScript IV First-Strand Synthesis System (Invitrogen) [125]. The qPCR assays were performed with SYBR Green PCR Master Mix in QuantStudio 3 (Applied Biosystems), primers used were listed in Supplementary Table S3 [126]. The 2^–ΔΔCt^ method was used to calculate relative gene expression after normalization to the β-actin internal control.

### Microelectrode arrays (MEA)

Organoids at around 21 days were seeded into 48-well transparent MEA plates (Axion Biosystems) at one organoid per well. MEA assays were performed with Maestro Pro platform (Axion Biosystems) and recorded using AxIS software. Organoids were fed with BrainPhys media (STEMCELL Technologies) supplemented with 1 × N2, 1 × B27, 20 ng/mL BDNF, 20 ng/mL GDNF, as well as brain extract or vehicle. For recording, following a 5 min resting time in the instrument, each plate was recorded for 10 min to calculate the spike per well. Experiments were repeated four times, each experiment used one hPSC line and contained 3–4 organoids. MEA analysis was performed using the Axion Biosystems NeuralMetrics Tool.

### Proteomics sample preparation and data analysis

Organoid proteins were extracted following a previously described pipeline with modifications [127]. Specifically, the organoids were solubilized using 1X RIPA buffer supplemented with 1X protease inhibitor and 1X phosphatase inhibitor. Next, the organoids tissue as disrupted in a tissue homogenizer and subjected to a 3x cycle sonication in a water bath. The protein concentration in the lysate was measured using a BCA assay. Proteins (~5 µg) were denatured by mixing with 4x LDS sample buffer and boiling at 95 °C for 10 min. The protein mixture was loaded into a 1 mm × 12-well NuPAGE^TM^ 12% Bis-Tris gel (ThermoFisher, NP0322BOX) and run at 200 V for 5 min using MOPS Running Buffer (ThermoFisher). Gel slices were placed in deionized water and washed on a rocker. The cleaned gel slices were proceeded to digest and extract peptides as described [128]. Proteins were digested with trypsin/LysC (Pierce^TM^) (1 µg trypsin/sample), and digested peptides were extracted and passed through Peptide Cleanup C18 Spin Tubes for desalting [128]. Desalted and dried peptides obtained from in-gel preparations were reconstituted in 5% (v/v) ACN and 0.1% (v/v) formic acid (FA) before being analyzed by HPLC-MS/MS using a 480-Exploris MS (ThermoFisher) equipped with an Ultimate 3000 HPLC (ThermoFisher) and an Aurora^TM^ Ultimate analytical column (IonOpticks). 500 ng of peptides for each sample were loaded on a trap column and separated on a 25 cm nanoflow UHPLC IonOpticks C18 column. Peptides were analyzed in a 130 min gradient of 12–45% buffer B (buffer A: 0.1% FA; buffer B: 80% ACN, 0.1% FA). All the data were acquired in a data-independent acquisition mode. The integrity and performance of the MS instrument were monitored using HeLa standards before and after the experimental runs.

MS raw data files were processed using Spectronaut software (version 18.4, Biognosys) using a Direct DIA mode. In silico spectral libraries were constructed using the latest version of the human proteome file downloaded from UniProt. The BGS factory settings were applied as a default for analyzing the data and selecting the candidates. The raw output files were subjected to multivariate analysis. Biognosys’s default package was used for the analysis and for sorting the DEPs using the criteria of FC ≥ 1.5, adjusted *p* < 0.05. The volcano plot was generated using default Biognosys settings. Pathway enrichment analysis was performed using the online tool Enrichr, with the Enrichr background library for the analysis [129]. We thank the Purdue Proteomics Facility for assistance with sample processing and data collection.

### Statistics and reproducibility

All data represent mean ± SEM. Significance is determined using a two-tailed unpaired t-test with Welch’s correction for comparing two independent groups, or a one-way ANOVA test with Bonferroni post-hoc test for comparing three or more groups. A *p* value < 0.05 was considered significant. Analyses were performed with GraphPad Prism 10. All experiments were independently performed at least three times.

## Supplementary information

Supplementary materials

## Data Availability

Supplementary information is accessible on MP’s website. The proteomics data have been deposited in ProteomeXchange under the accession code MSV000096777. Additional datasets and materials can be obtained by contacting the corresponding author (R.X.) upon request.
