# Supplementary material for: Alzheimer’s disease patient brain extracts induce multiple pathologies in novel vascularized neuroimmune organoids for disease modeling and drug discovery
Source: Mol Psychiatry. 2025 May 2;30(10):4558–75. doi: 10.1038/s41380-025-03041-w (PMC12436168; doi:10.1038/s41380-025-03041-w)
Supplement: Supplementary file 1 — Supplementary materials [file 41380_2025_3041_MOESM1_ESM.docx]

**Supplementary Figures and Tables**

**Figure S1. Representative images of sections of organoids with CAGG hPSC-derived vasculatures.**

(A) Vasculature formation in whole organoids, scale bar, 400 μm.

(B) Local magnification of blood vessel tubular structure, scale bar, 200 μm.

(C) Co-locolization of collagen IV^+^and CAGG GFP^+^ signals, scale bars, 20 μm or 10 μm as indicated.

(D) Co-locolization of PDGFRβ^+^ and CAGG GFP^+^ signals, scale bars, 20 μm or 10 μm as indicated.


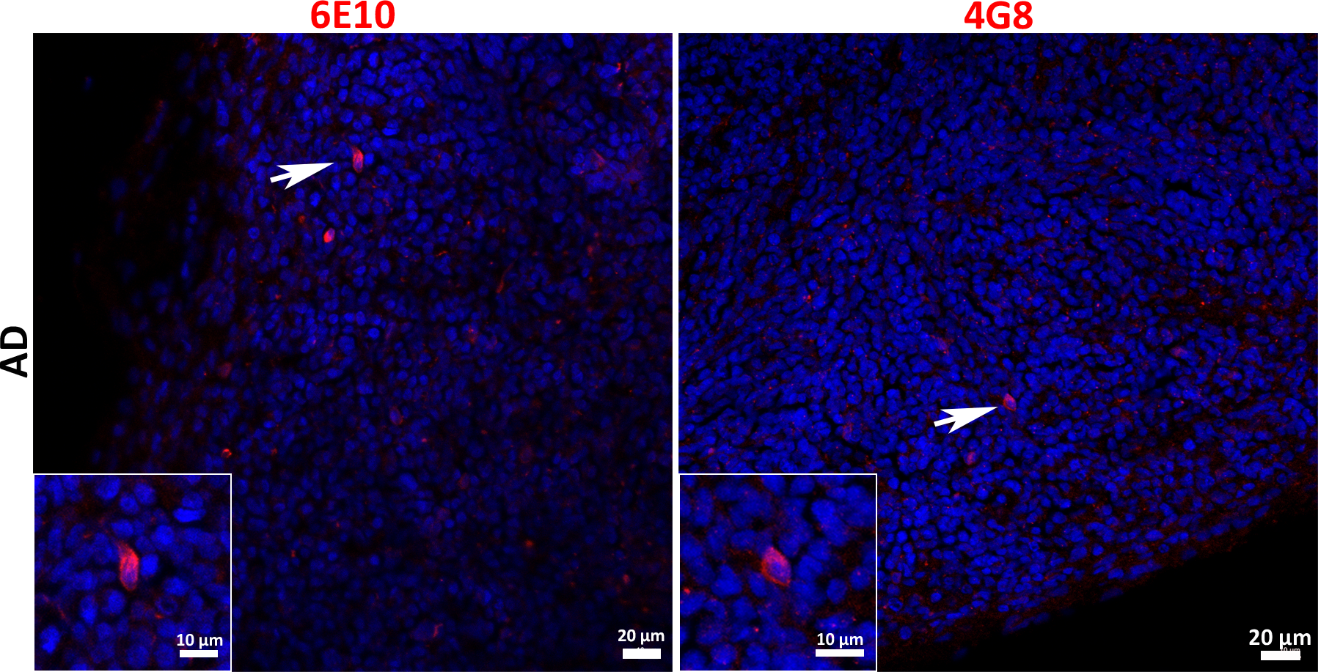


**Figure S2. Diffused 6E10 and 4G8 signals in organoids at 2 weeks post-exposure to AD brain extracts**, **indicating endogenous APP instead of Aβ aggregates.** Scale bars, 20 or 10 μm as indicated.

**
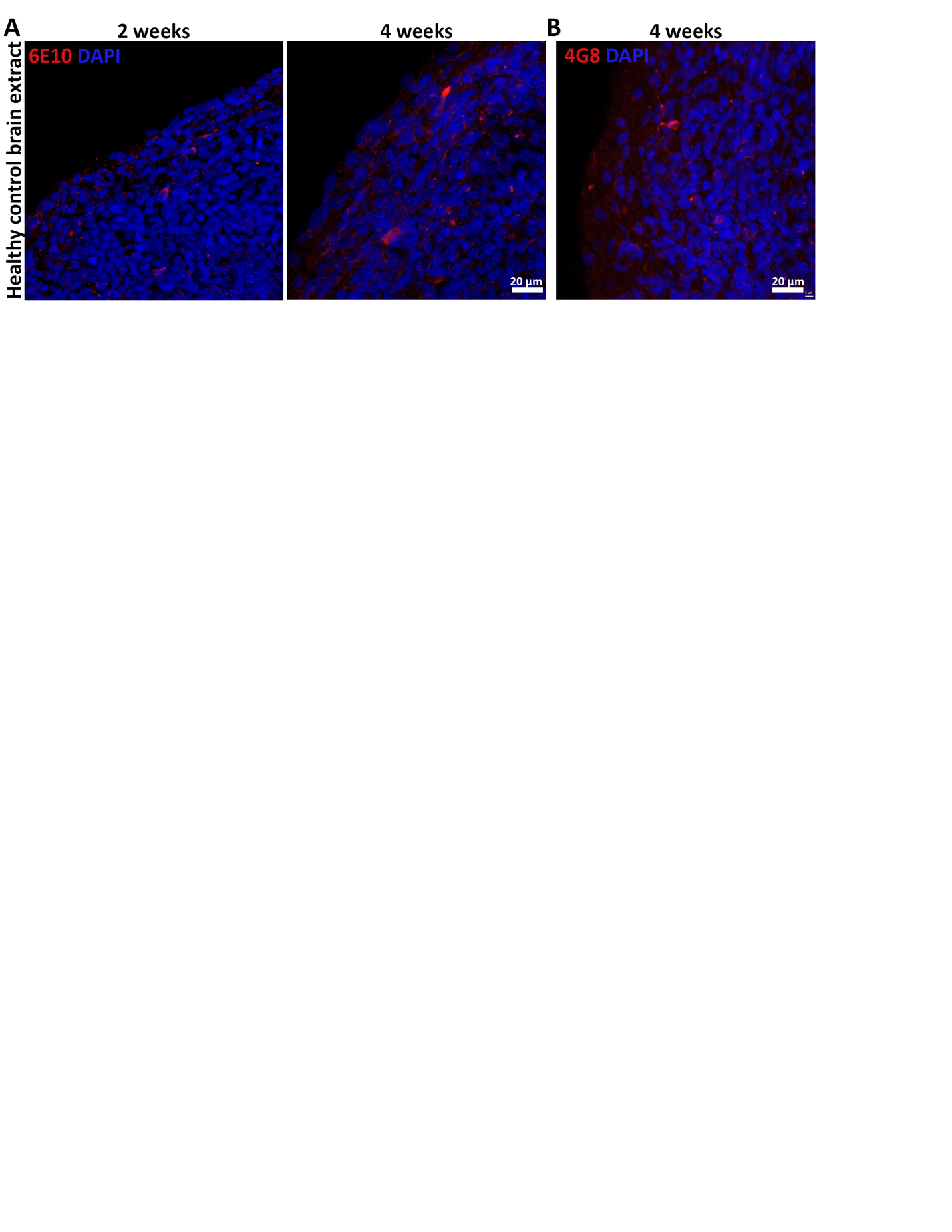
**

**Figure S3. Diffuse but not aggregated** **6E10 and 4G8 signals in organoids treated with healthy individual-derived brain extracts.**

(A) Diffuse 6E10-positive signals observed in organoids at 2 weeks or 4 weeks post-exposure to age-matched healthy individual-derived brain extracts (healthy control brain extract). Scale bar, 20 μm.

(B) Diffuse 4G8-positive signals observed in organoids at 4 weeks post-exposure to brain extracts from age-matched healthy individuals (healthy control brain extract). Scale bar, 20 μm.

**
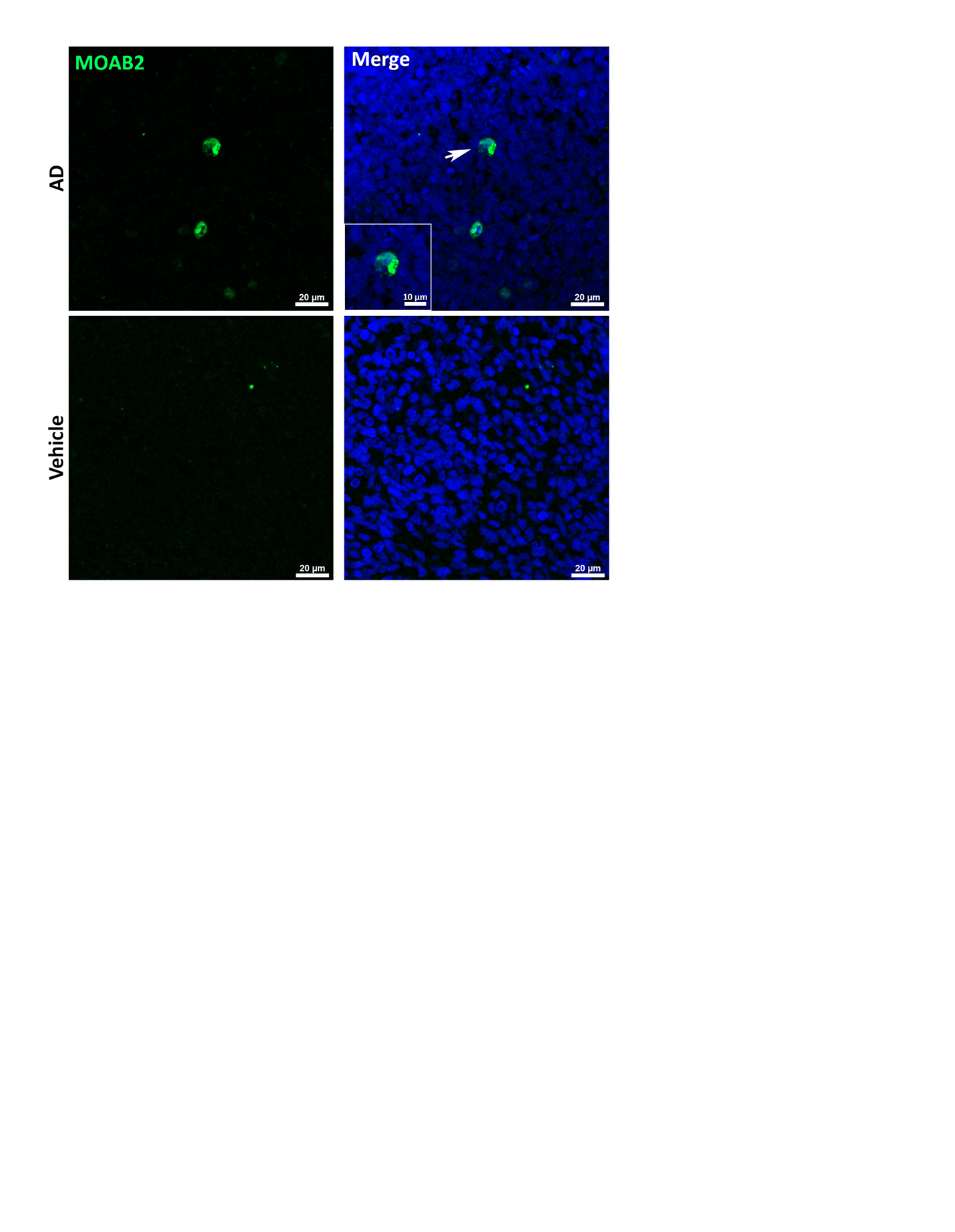
**

**Figure S4. Organoids exposed to AD brain extracts but not the vehicle showed intraneuronal Aβ aggregates.**

Representatives of MOAB2^+^ cells in organoids at 2 weeks post-exposure to AD brain extracts or vehicle. Scale bars, 20 μm.


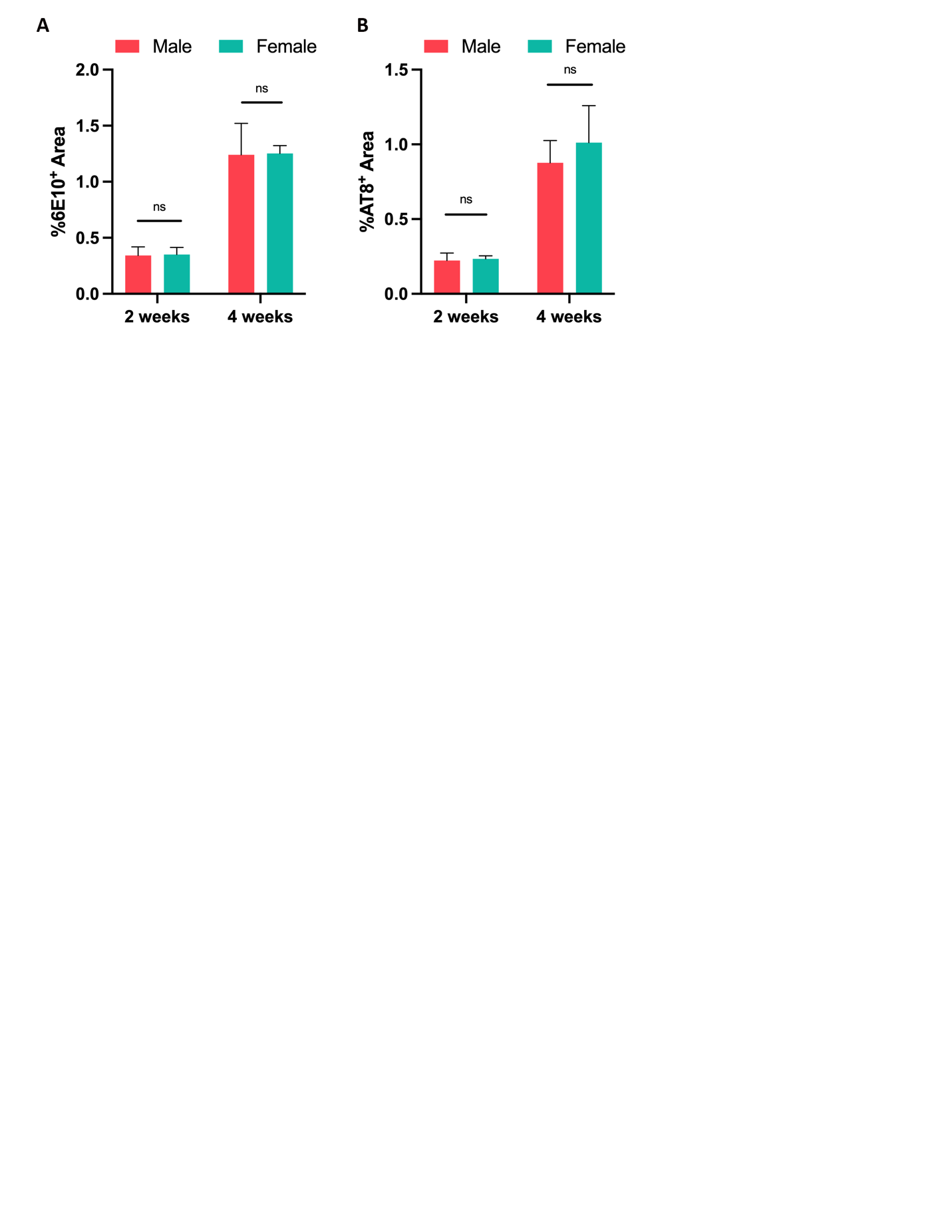


**Figure S5. Quantification of 6E10^+^ and AT8^+^ Area from organoids generated from male and female hPSC lines.** After 2 weeks or 4 weeks of brain extract exposure, %6E10^+^ Area (A) and % AT8^+^ Area (B) were quantified in both male and female hiPSC-derived organoids. For the male group, n=4 independent experiments from 4 different cell lines. Each experiment used one hPSC cell line and contained 4-6 organoids. For the female group, n=3 independent experiments from 1 cell line. Each experiment contained 4 organoids. Data are represented as Mean ± SEM. Unpaired t test with Welch’s correction, ns represents no significance.


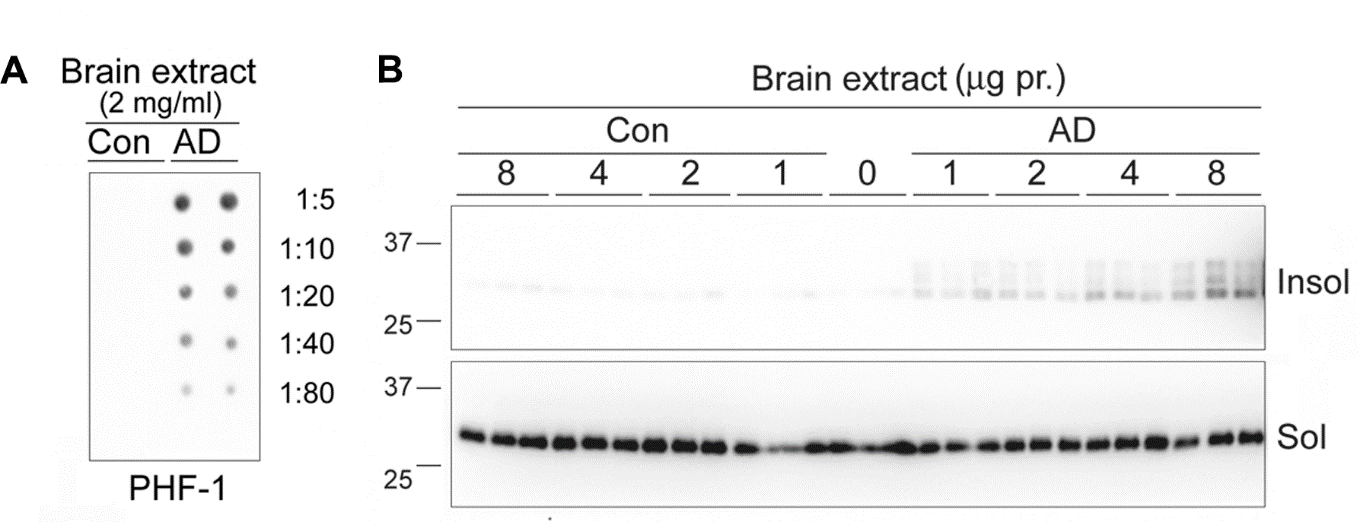


**Figure S6. Confirming tau seeding activity of AD and age-matched healthy individual (Con)-derived brain extracts.**

(A) Representative image showing PHF-1 (pS396/pS404) immunoreactivity in brain extracts.

(B) Confirmation of tau seeding activity in brain extracts presented by insoluble tau using seeded tau aggregation assay, and AD but not Con brain extracts show tau seeding activity.


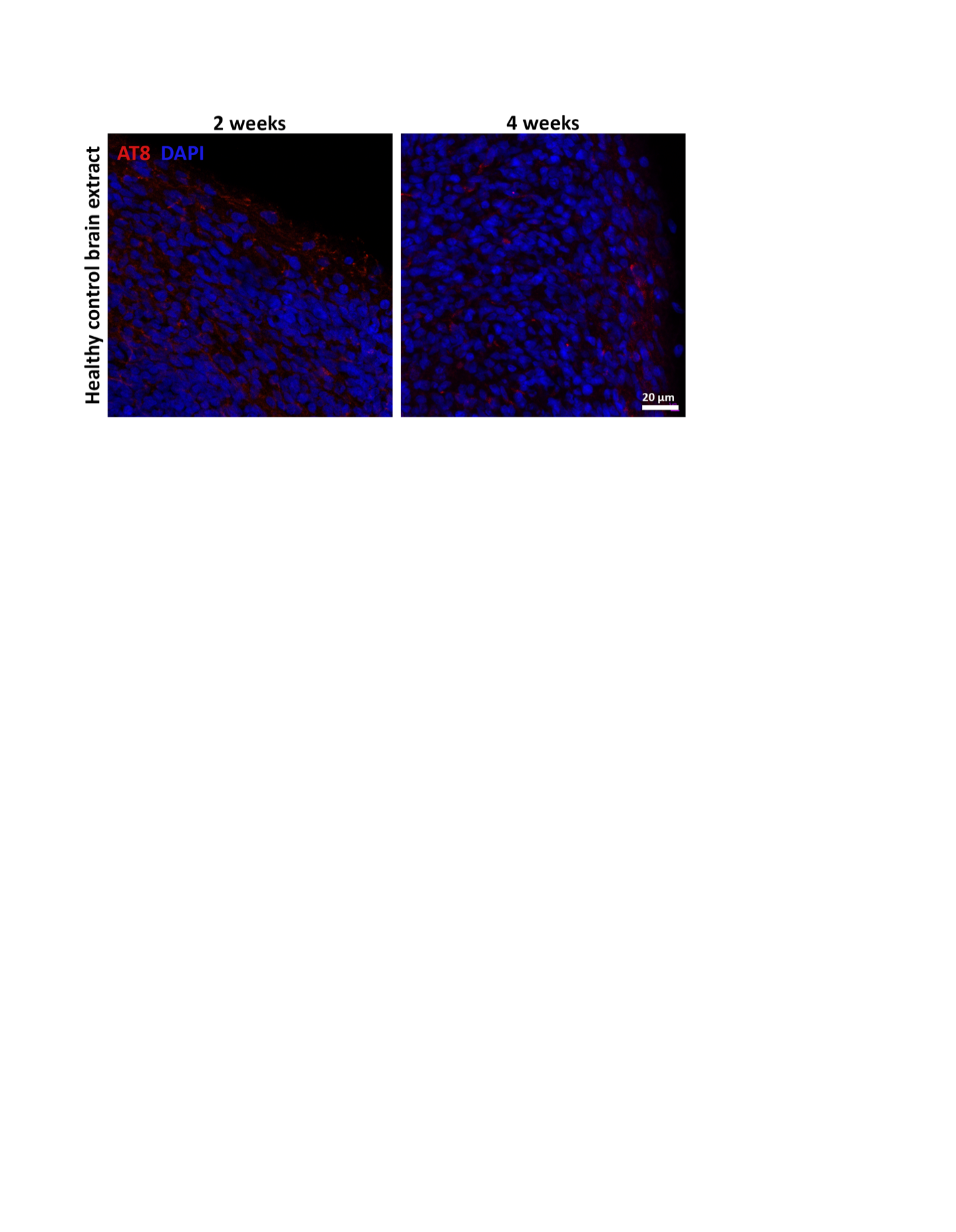


**Figure S7. AT8 signals in organoids treated with healthy individual-derived brain extract.** Diffuse AT8^+^ signals were observed in organoids at 2 weeks post-exposure to healthy individual-derived brain extracts, but no AT8^+^ signal was observed at 4 weeks post-exposure. Scale bar, 20 μm.


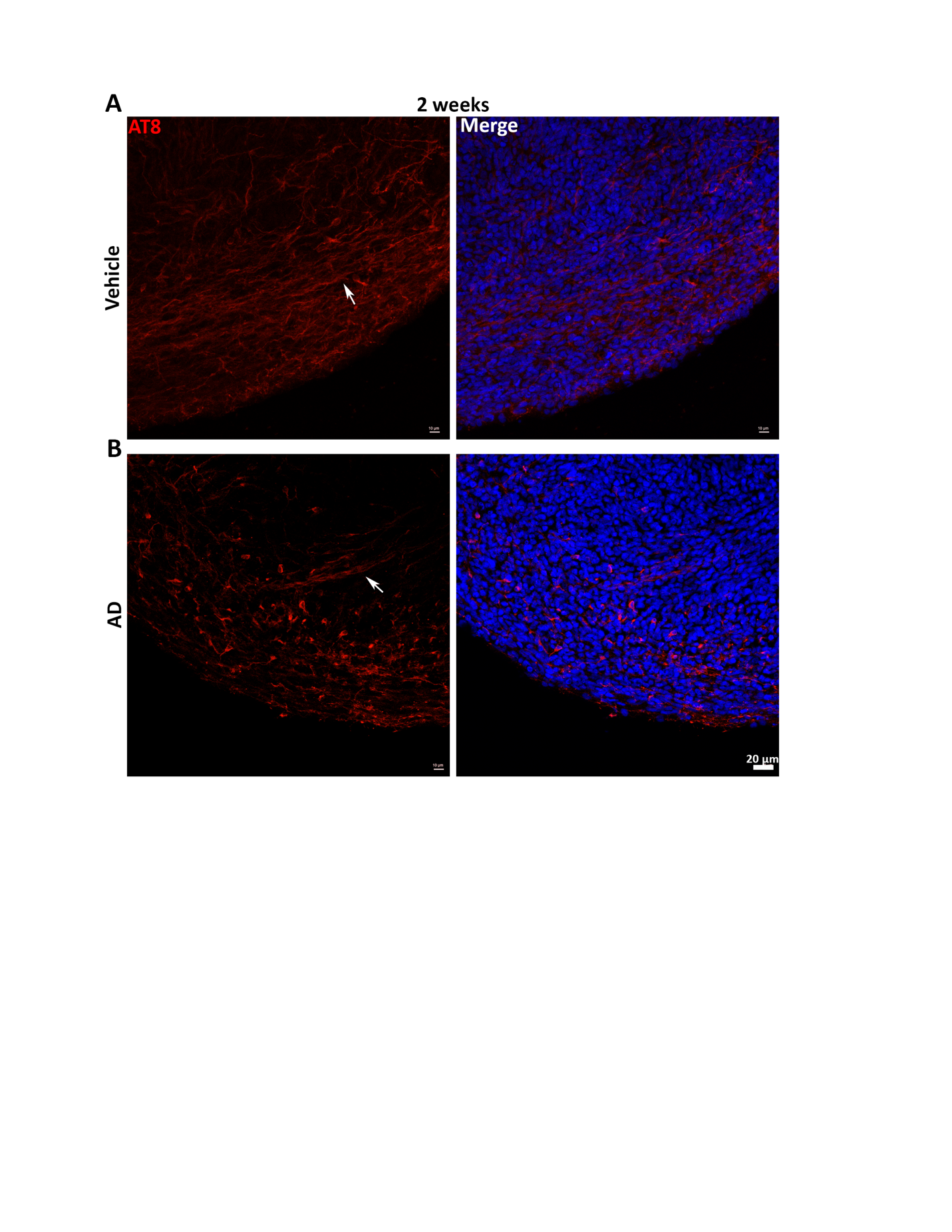


**Figure S8. AT8 signals in organoids at 2 weeks post-exposure to the vehicle or AD brain extracts.**

(A)  Representative image showing only modest diffuse axonal AT8^+^ signals in organoids at 2 weeks post-exposure to vehicle. Diffuse AT8 signals were pointed with arrows. Scale bars, 20 μm.

(B) Representative image showing AT8^+^ signals in organoids at 2 weeks post-exposure to AD brain extracts. Mainly aggregation was observed while few diffuse axonal signals were captured. Diffuse AT8 signals were pointed with arrows. Scale bars, 20 μm.


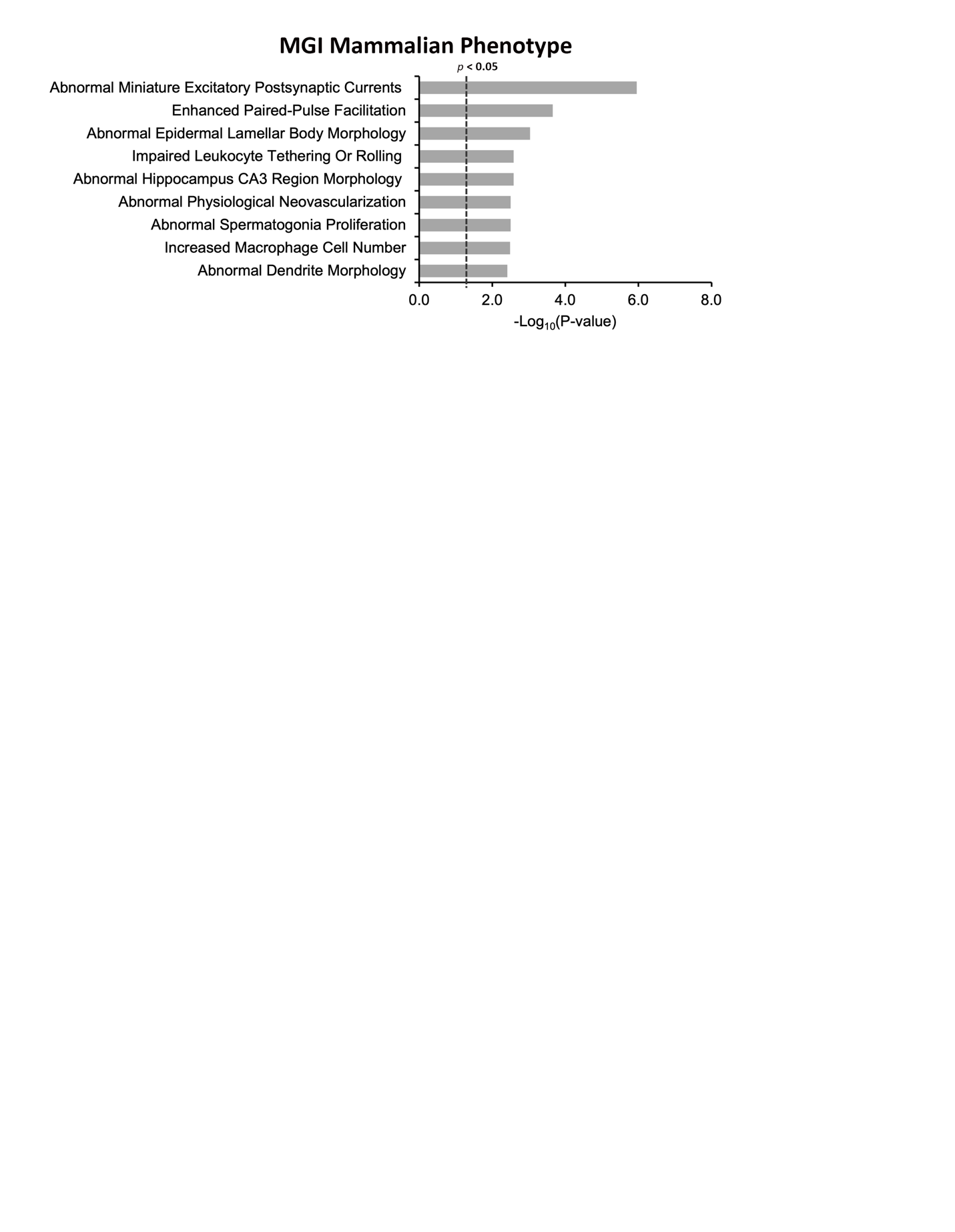


**Figure S9. MGI mammalian phenotype enrichment analysis of upregulated DEPs between AD group and vehicle group organoids.**


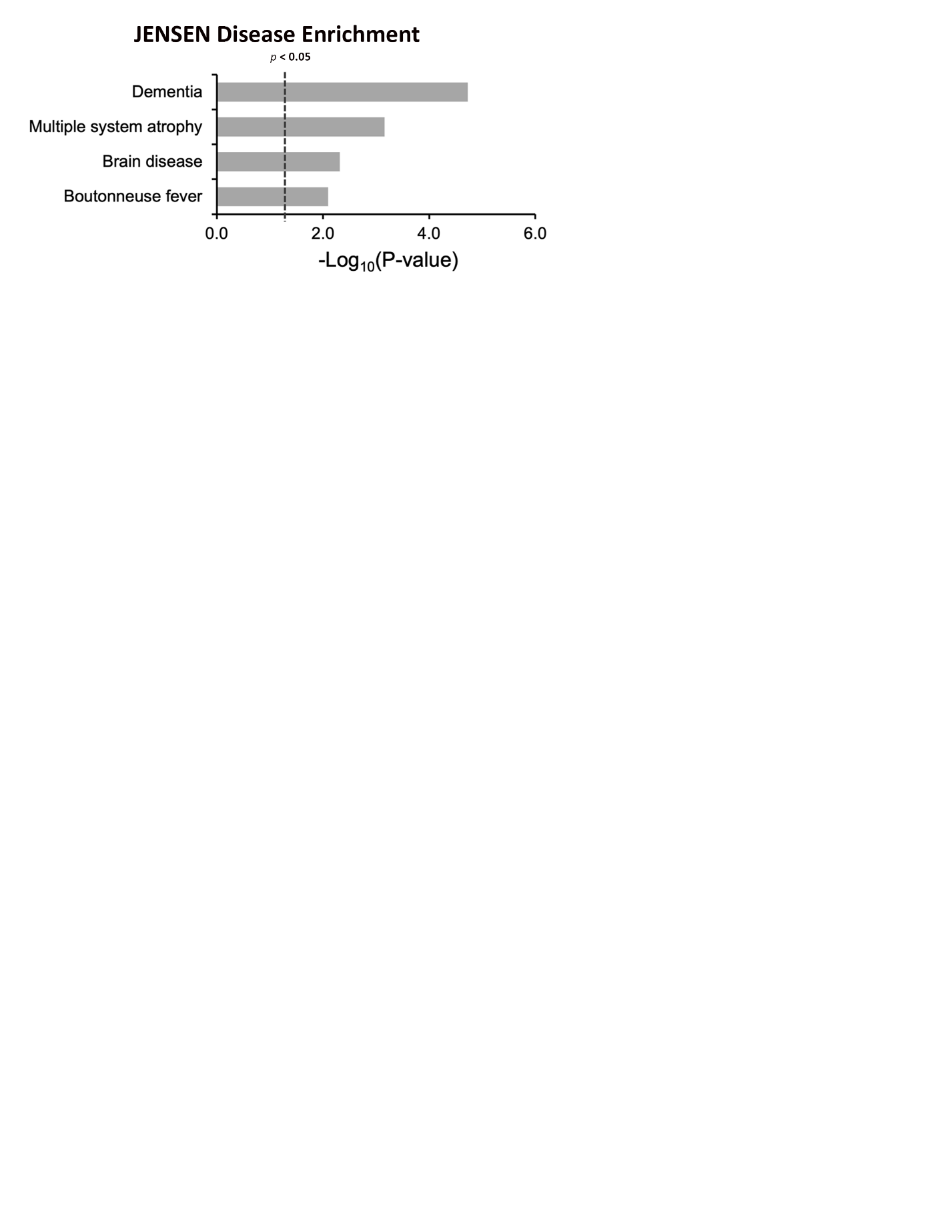


**Figure S10. JENSEN Disease Enrichment analysis of 28 DEPs consistent with patient brain tissue derived results.** We identified 28 DEPs that have a consistent upregulating trend with proteomics data obtained from pre-clinical/mild cognitive impairment/clinical stage AD patients.

**Tabel S1.** Cell lines used to generate NPCs, PMPs, and VPs.

| **Cell line** | **Gender** | **Donor** | **APOE allele** | **Type** | **Source** |
| --- | --- | --- | --- | --- | --- |
| KOLF 2.1 | Male | healthy | 3/3 | hiPSC | The Jackson Laboratory |
| ND2.0 | Male | healthy | 3/3 | hiPSC | NIH |
| GCaMP (KOLF2.1 derived) | Male | healthy | 3/3 | hiPSC | The Jackson Laboratory |
| UTY1 | Female | healthy | 3/3 | hiPSC | NIH |
| ApoE4/4 (KOLF2.1 C112R) | Male | healthy | 4/4 | hiPSC | The Jackson Laboratory |
| CAGG | Male | healthy | 3/3 | hESC | NIH |

**Table S2.** Antibodies used for immunostaining.

| **Antibodies** | **Source** | **Dilution** |
| --- | --- | --- |
| Aβ42 | Thermo, Invitrogen H31L21 | 1:1000 |
| AT8 | Invitrogen, MN1020 | 1:1000 |
| βIII-tubulin | Millipore, MAB1637MI | 1:200 |
| CD235 | Thermo, Invitrogen PA5-27154 | 1:200 |
| CD43 | Thermo, Invitrogen 14-0439-82 | 1:100 |
| CD31 | R&D, AF3628 | 1:20 |
| CD45 | Thermo, Invitrogen 14-9457-82 | 1:40 |
| Caspase3 | Cell Signaling Technology, Asp175 | 1:2000 |
| Collagen IV | NOVUS, NB120-6586SS | 1:500 |
| Homer1 | SYSY, 160003 | 1:200 |
| IBA1 | Wako, Cat. #019-19741 | 1:1000 |
| MAP2 | Santa Cruz, sc-74420 | 1:100 |
| MOAB2 | Novus Biologicals, NBP2-13075 | 1:1000 |
| NESTIN | R&D, MAB1259 | 1:200 |
| NeuN | Millipore, ABN78 | 1:500 |
| PAX6 | GeneTex, GTX113241 | 1:200 |
| PDGFRβ | Cell Signaling Technology, | 1:40 |
| PHF-1 | From Dr. Peter Davies | 1:500 |
| pThr217 | Cell signaling Technology, #51625 | 1:200 |
| S100β | Sigma, S2532 | 1:200 |
| 6E10 | Biolegend, 803001 | 1:500 |
| 4G8 | Biolegend, 800701 | 1:500 |

**Table S3.** Sequence of primers used in qPCR.

|  | **Forward primer** | **Reverse primer** |
| --- | --- | --- |
| **Human IL6** | ACTCACCTCTTCAGAACGAATTG | CCATCTTTGGAAGGTTCAGGTTG |
| **Human CCL2** | CAGCCAGATGCAATCAATGCC | TGGAATCCTGAACCCACTTCT |
| **Human VCAM-1** | GTCTCCAATCTGAGCAGCAA | TGGGAAAAACAGAAAAGAGGTG |
| **Human VE-Cadherin** | AAGGACATAACACCACGAAACG | CAAACTGCCCATACTTGACTGTG |
| **Human β actin** | CATGTACGTTGCTATCCAGGC | CTCCTTAATGTCACGCACGAT |

**Table S4.** Upregulated proteins in AD group organoids compared with control group

| NDUFAB1 |
| --- |
| VRK1 |
| CCDC167 |
| NDRG2 |
| UQCC6 |
| TMEM59 |
| IFT46 |
| DISP2 |
| SNCB |
| MPZL1 |
| SSR1 |
| VSNL1 |
| H1-0 |
| CD63 |
| IFITM3 |
| NHSL1 |
| IGFBP2 |
| MTO1 |
| CD320 |
| DCTPP1 |
| CREG1 |
| COQ9 |
| MICOS13 |
| SS18L1 |
| ACTN4 |
| CD44 |
| S100A16 |
| TSPAN3 |
| FXYD6 |
| CCDC90B |
| NFYB |
| H2AZ1 |
| GFAP |
| CD82 |
| SNRPC |
| EPDR1 |
| PEA15 |
| H1-5 |
| SNCA |
| ECE1 |
| CD9 |
| TMED9 |
| SYT3 |
| BET1L |
| FAM162A |
| OR5AC2 |
| MANF |
| CNN2 |
| WASF2 |
| VTI1A |
| AMOTL1 |
| TGOLN2 |
| CIRBP |
| SNAP29 |
| RNF13 |
| SNAP25 |
| ATP6V0D2 |
| ATOX1 |
| BNIP3L |
| MAPT |
| HMGB2 |
| LOC122394732 |
| COX6B1 |
| H1-4 |
| H1-2 |
| HPCAL1 |
| VAMP2 |
| NDUFA7 |
| H1-3 |
| TOM1L2 |
| EFCAB14 |
| TTC33 |
| PAFAH1B2 |
| CD200 |
| SPATA33 |
| TMCC2 |

**Table S5.** Downregulated proteins in AD group organoids compared with control group

| ECD |
| --- |
| FGFBP3 |
| HSP90AB4P |
| FAM200C |
| SLC25A17 |
| LRRCC1 |
| RPS4Y1 |
| TGFBRAP1 |
| SLC19A1 |
| TH |
| TMEM70 |

**Table S6.** Upregulated proteins in AD organoids consistent with patient data

| FXYD6 |
| --- |
| COQ9 |
| CD82 |
| ECE1 |
| NDRG2 |
| VTI1A |
| CD200 |
| S100A16 |
| SNAP29 |
| CNN2 |
| TTC33 |
| H1-4 |
| H1-2 |
| EPDR1 |
| VSNL1 |
| MAPT |
| H1-3 |
| H1-5 |
| CD9 |
| HMGB2 |
| COX6B1 |
| PEA15 |
| CREG1 |
| PAFAH1B2 |
| GFAP |
| H1-0 |
| SNCB |
